# The Cryptic Plastid of *Euglena longa* Defines a New Type of Nonphotosynthetic Plastid Organelle

**DOI:** 10.1128/mSphere.00675-20

**Published:** 2020-10-21

**Authors:** Zoltán Füssy, Kristína Záhonová, Aleš Tomčala, Juraj Krajčovič, Vyacheslav Yurchenko, Miroslav Oborník, Marek Eliáš

**Affiliations:** a Institute of Parasitology, Biology Centre ASCR, České Budějovice, Czech Republic; b Faculty of Science, Charles University, BIOCEV, Vestec, Czech Republic; c Faculty of Science, University of South Bohemia, České Budějovice, Czech Republic; d Life Science Research Centre, Department of Biology and Ecology, Faculty of Science, University of Ostrava, Ostrava, Czech Republic; e Institute of Environmental Technologies, Faculty of Science, University of Ostrava, Ostrava, Czech Republic; f Department of Biology, Faculty of Natural Sciences, University of Ss. Cyril and Methodius in Trnava, Trnava, Slovakia; Aix-Marseille University

**Keywords:** Calvin-Benson cycle, *Euglena longa*, Euglenophyceae, evolution, nonphotosynthetic plastids, phylloquinone, redox balance, sulfoquinovosyldiacylglycerol, tocopherol

## Abstract

Most secondary nonphotosynthetic eukaryotes have retained residual plastids whose physiological role is often still unknown. One such example is Euglena longa, a close nonphotosynthetic relative of Euglena gracilis harboring a plastid organelle of enigmatic function. By mining transcriptome data from E. longa, we finally provide an overview of metabolic processes localized to its elusive plastid. The organelle plays no role in the biosynthesis of isoprenoid precursors and fatty acids and has a very limited repertoire of pathways concerning nitrogen-containing metabolites. In contrast, the synthesis of phospholipids and glycolipids has been preserved, curiously with the last step of sulfoquinovosyldiacylglycerol synthesis being catalyzed by the SqdX form of an enzyme so far known only from bacteria. Notably, we show that the *E. longa* plastid synthesizes tocopherols and a phylloquinone derivative, the first such report for nonphotosynthetic plastids studied so far. The most striking attribute of the organelle could be the presence of a linearized Calvin-Benson (CB) pathway, including RuBisCO yet lacking the gluconeogenetic part of the standard cycle, together with ferredoxin-NADP^+^ reductase (FNR) and the ferredoxin/thioredoxin system. We hypothesize that the ferredoxin/thioredoxin system activates the linear CB pathway in response to the redox status of the *E. longa* cell and speculate on the role of the pathway in keeping the redox balance of the cell. Altogether, the *E. longa* plastid defines a new class of relic plastids that is drastically different from the best-studied organelle of this category, the apicoplast.

**IMPORTANCE** Colorless plastids incapable of photosynthesis evolved in many plant and algal groups, but what functions they perform is still unknown in many cases. Here, we study the elusive plastid of *Euglena longa*, a nonphotosynthetic cousin of the familiar green flagellate Euglena gracilis. We document an unprecedented combination of metabolic functions that the *E. longa* plastid exhibits in comparison with previously characterized nonphotosynthetic plastids. For example, and truly surprisingly, it has retained the synthesis of tocopherols (vitamin E) and a phylloquinone (vitamin K) derivative. In addition, we offer a possible solution of the long-standing conundrum of the presence of the CO_2_-fixing enzyme RuBisCO in *E. longa*. Our work provides a detailed account on a unique variant of relic plastids, the first among nonphotosynthetic plastids that evolved by secondary endosymbiosis from a green algal ancestor, and suggests that it has persisted for reasons not previously considered in relation to nonphotosynthetic plastids.

## Abstract (precis)

Colorless plastids incapable of photosynthesis evolved in many plant and algal groups, but what functions they perform is still unknown in many cases. Here, we study the elusive plastid of *Euglena longa*, a nonphotosynthetic cousin of the familiar green flagellate Euglena gracilis. We document an unprecedented combination of metabolic functions that the *E. longa* plastid exhibits in comparison with previously characterized nonphotosynthetic plastids. For example, and truly surprisingly, it has retained the synthesis of tocopherols (vitamin E) and a phylloquinone (vitamin K) derivative. In addition, we offer a possible solution of the long-standing conundrum of the presence of the CO_2_-fixing enzyme RuBisCO in *E. longa*. Our work provides a detailed account on a unique variant of relic plastids, the first among nonphotosynthetic plastids that evolved by secondary endosymbiosis from a green algal ancestor, and suggests that it has persisted for reasons not previously considered in relation to nonphotosynthetic plastids.

## INTRODUCTION

Photosynthesis was supposedly the primary evolutionary advantage driving the acquisition of the primary plastid as well as its further spread in eukaryotes by secondary and higher-order endosymbioses (1–3). However, plastids host many other metabolic pathways, such as biosynthesis of amino and fatty acids, isopentenyl pyrophosphate (IPP) and its derivatives (isoprenoids), and tetrapyrroles (4–6). Hence, reversion of photosynthetic lineages to heterotrophy typically does not entail plastid loss, and nonphotosynthetic plastids are found in many taxa (7–10).

The most extensively studied relic plastid is the apicoplast of apicomplexan parasites (Plasmodium falciparum and Toxoplasma gondii, above all). The essentiality of the apicoplast for parasite survival has attracted much attention as a promising target for parasite-specific inhibitors (11, 12). So far, three plastid pathways seem to be the reason for the apicoplast retention: non-mevalonate IPP synthesis, heme synthesis, and type II fatty acid synthesis (FASII) (13). Less is known about plastid metabolic functions in other nonphotosynthetic algal lineages. Many of them have a metabolic capacity similar to that of the apicoplast (10, 14, 15), but some house a more complex metabolism that includes amino acid biosynthesis and carbohydrate metabolism pathways (16–18). Until recently, IPP synthesis appeared to be a process conserved even in the most reduced plastids, such as the genome-lacking plastids of certain alveolates (8, 19). However, nonphotosynthetic plastids lacking this pathway have now been documented (9, 20, 21). Thus, there generally is a metabolic reason for plastid retention, although the cases of plastid dependency differ between lineages.

Like their prime representative Euglena gracilis, most euglenophytes are mixotrophs containing complex three-membrane-bound plastids derived from a green alga (22–24). Nonphotosynthetic mutants of E. gracilis are capable of heterotrophic living (reviewed in references 7 and 25), and several euglenophyte lineages independently became secondarily heterotrophic (26). The best known is *Euglena* (previously *Astasia*) *longa*, a close relative of E. gracilis (26, 27). Although documentation at the cytological level is doubtful (28–30), molecular sequence data provide clear evidence for the presence of a cryptic plastid organelle in this species. The E. longa plastid genome was sequenced 2 decades ago (31) and was shown to lack any photosynthesis-related genes, surprisingly except for *rbcL* encoding the large subunit of ribulose-1,5-bisphosphate carboxylase/oxygenase (RuBisCO). More recently, the existence of a nuclear-gene-encoded small RuBisCO subunit (RBCS), synthesized as a precursor polyprotein, was documented in *E. longa*, although its processing into monomers could not be demonstrated (32). The physiological role of the *E. longa* RuBisCO and the whole plastid remains unknown, but indirect evidence suggests that the plastid is essential for the survival of *E. longa* (33–36).

To provide a resource for investigating the biology of *E. longa* and its plastid, we generated a transcriptome assembly and demonstrated its high completeness and utility (37). We also showed that nuclear-gene-encoded plastidial proteins in *E. longa* employ an N-terminal plastid-targeting bipartite topogenic signal (BTS) of the same two characteristic classes known from E. gracilis. The *E. longa* transcriptome revealed unusual features of the plastid biogenesis machinery shared with photosynthetic euglenophytes but also suggested specific reductions of housekeeping functions, reflecting the loss of photosynthesis (37). Nevertheless, the anabolic and catabolic pathways localized to the *E. longa* colorless plastid have not been characterized. Hence, we set out to exploit the available sequence data to chart the metabolic map of the *E. longa* plastid. The analyses were greatly facilitated by the recent characterization of the E. gracilis plastid metabolic network based on a proteomic analysis of the organelle (38). Our study provides the first comprehensive view of a nonphotosynthetic secondary plastid of green algal origin and shows that the metabolic capacity of the *E. longa* plastid is strikingly different from those of the apicoplast and other relic plastids characterized in sufficient detail.

## RESULTS

### The plastid protein complement of *E. longa* is dramatically reduced compared to that of its photosynthetic cousin.

To obtain a global view of the repertoire of the plastid proteins in *E. longa*, we searched its transcriptome assembly to identify putative orthologs of the proteins defined as part of the E. gracilis plastid proteome (38). Of the 1,312 such proteins encoded by the E. gracilis nuclear genome, less than half (594) exhibited an *E. longa* transcript that met our criteria for orthology (see Data Set S1, tab 1, in the supplemental material). As expected, the functional categories with the least proportion of putative *E. longa* orthologs included “photosynthesis,” “metabolism of cofactors and vitamins,” and “reaction to oxidative and toxic stress,” with 95.89%, 85.11%, and 73.33% of the proteins missing in *E. longa*, respectively. Interestingly, *E. longa* also lacks counterparts of some plastidial proteins involved in gene expression and genome maintenance, suggesting that the metabolic simplification, primarily the loss of photosynthesis itself with its high demand on protein turnover and mutagenic effects on the plastid genome, may have relaxed the constraints on the respective housekeeping molecular machineries.

10.1128/mSphere.00675-20.8 DATA SET S1 Supplemental tables (tab 1 to tab 22). Download Data Set S1, XLSX file, 0.7 MB. Copyright © 2020 Füssy et al. 2020 Füssy et al. This content is distributed under the terms of the Creative Commons Attribution 4.0 International license.

Although these results clearly demonstrate the dramatic reduction of the functional complexity of the *E. longa* plastid compared to the plastid of its photosynthetic relative, they should not be interpreted such that the plastid harbors exactly the ∼600 proteins identified by the orthology search. First, the proteomically defined set of the putative E. gracilis plastid proteins is certainly affected by the presence of false-negative results (bona fide plastid proteins missed by the analysis) as well as false-positive results (contaminants) (38). Second, orthology does not necessarily imply the same subcellular localization. Hence, to obtain a finer view of the physiological functions of the *E. longa* plastid, we systematically searched for homologs of enzymes underpinning metabolic pathways known from plastids in general. N-terminal regions of the candidates were evaluated for characteristics of presequences predicting a specific subcellular localization to distinguish those likely representing plastid-targeted proteins from enzymes located in other compartments. Some of the bioinformatic predictions were further tested by biochemical analyses.

### The *E. longa* plastid lacks the MEP pathway of IPP biosynthesis yet has kept the production of tocopherol and a phylloquinone derivative.

**FIG 1:**
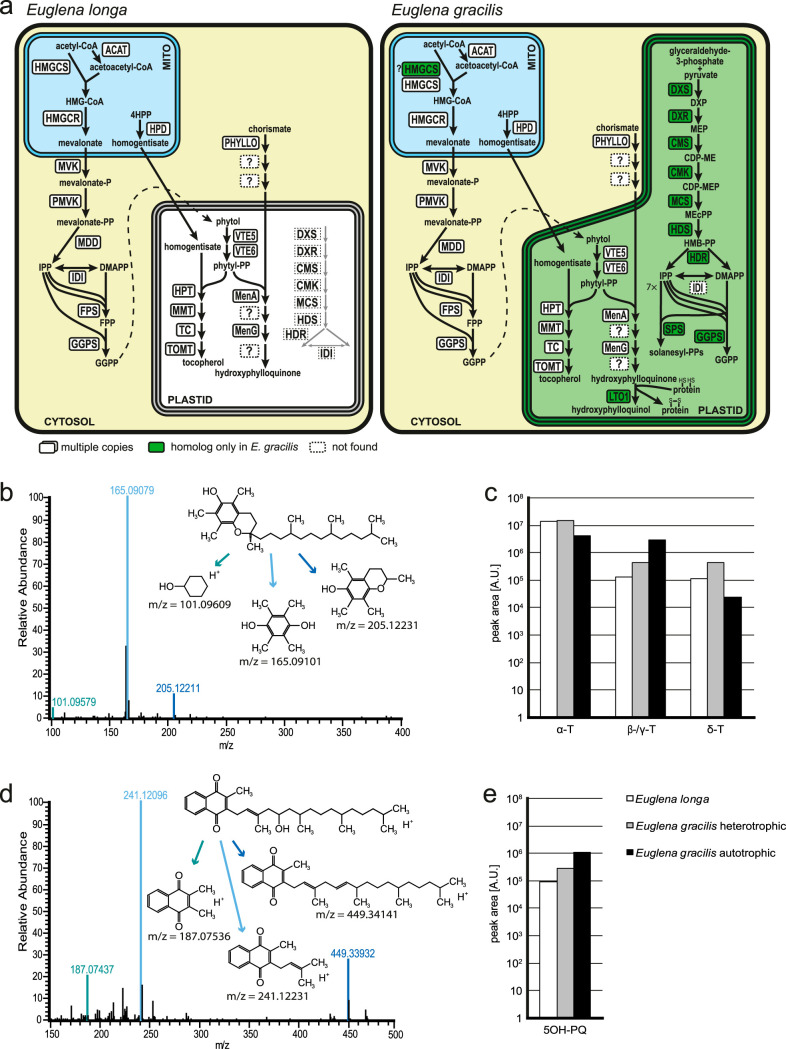
IPP and terpenoid-quinone biosynthesis in *E. longa* and its phototrophic relative E. gracilis. (a) Schematic comparison of the localization of enzymes (see the key below the *E. longa* “cell”). A question mark indicates unknown molecular identity of some of the enzymes (whose subcellular localization suggested by the figure must be considered tentative). Abbreviations for IPP synthesis: ACAT, acetyl-CoA acetyltransferase; CDP-ME, 4-(cytidine 5′-diphospho)-2-C-methyl-d-erythritol; CDP-MEP, 2-phospho-CDP-ME; CMK, CDP-ME kinase; CMS, CDP-ME synthase; DMAPP, dimethylallyl diphosphate; DXP, 1-deoxy-d-xylulose 5-phosphate; DXR, DXP reductase; DXS, DXP synthase; FPP, farnesyl diphosphate synthase; GGPS, geranylgeranyl-diphosphate synthase; HDR, HMB-PP reductase; HDS, HMB-PP synthase; HMB-PP, 4-hydroxy-3-methylbut-2-en-1-yl diphosphate; HMG-CoA, 3-hydroxy-3-methylglutaryl-CoA; HMGCR, HMG-CoA reductase; HMGCS, HMG-CoA synthase; IDI, isopentenyl-diphosphate delta-isomerase; MCS, MEcPP synthase; MDD, mevalonate-diphosphate decarboxylase; MEcPP, 2-C-methyl-d-erythritol 2;4-cyclodiphosphate; MEP, 2-C-methyl-d-erythritol 4-phosphate; MVK, mevalonate kinase; PMVK, phosphomevalonate kinase; PPS, unspecified polyprenyl-diphosphate synthase; ?, unclear substrate or unknown enzyme. Abbreviations for terpenoid-quinone synthesis: 4HPP, 4-hydroxyphenylpyruvate; HPD, hydroxyphenylpyruvate dioxygenase; HPT, homogentisate phytyltransferase; LTO1, PhQ-reducing oxidoreductase; MMT, MPBQ/MPSQ methyltransferase; TC, tocopherol cyclase; TOMT, tocopherol-*O*-methyltransferase; VTE5, phytyl kinase; VTE6, phytyl-phosphate kinase. (b) MS/MS spectrum record of *E. longa* α-tocopherol and the proposed fragmentation pattern in positive ionization mode (inset). Monoisotopic masses of particular fragments were obtained by simulation in the Xcalibur software. (c) Semiquantitative comparison of tocopherol species in *E. longa*, heterotrophically (dark) grown E. gracilis, and autotrophically grown E. gracilis. Peak area is shown in arbitrary units (A.U.) (see the legend to Fig. 2). (d and e) MS/MS spectrum record of *E. longa* 5-hydroxyphylloquinone and the proposed fragmentation pattern in positive ionization mode (inset); semiquantitative comparison of 5-hydroxyphylloquinone in *E. longa*, heterotrophically (dark) grown E. gracilis, and autotrophically grown E. gracilis.

There are two parallel pathways of IPP biosynthesis in E. gracilis (39): the mevalonate (MVA) pathway localized to the mitochondrion (first three enzymes) and the cytosol (the rest), and the plastid-localized 2-C-*methyl*-d-erythritol (MEP) pathway, the latter providing precursors for synthesis of terpenoid compounds connected to photosynthesis, namely, carotenoids and plastoquinone (38, 39). As expected, only enzymes of the MVA pathway were found in *E. longa* (Data Set S1, tab 2, and Fig. 1a). The carotenoid and plastoquinone biosynthesis enzymes are all missing, but surprisingly, the *E. longa* plastid appears to still be involved in terpenoid metabolism, specifically in its phytol branch.

Photosynthetic eukaryotes generally produce three types of phytol derivatives, tocopherols (vitamin E), phylloquinone (PhQ; vitamin K_1_) and chlorophyll, starting with a common precursor phytyl diphosphate (phytyl-PP), which is (directly or indirectly via salvage of phytol liberated by chlorophyll degradation) made by reduction of geranylgeranyl-PP derived from the MEP pathway (40). E. gracilis has proven to be unusual not only because it lacks the conventional geranylgeranyl-PP reductase (38), but also for making phytol from a precursor provided by the MVA pathway (39, 41). The route of phytol synthesis is currently unknown, though phytyl-PP might be synthesized in the E. gracilis plastid exclusively by the stepwise phosphorylation of phytol by phytol kinase (VTE5) and phytyl phosphate kinase (VTE6), enzymes employed in plants in phytol salvage (38). *E. longa* has retained both VTE5 and VTE6, each being highly similar to their E. gracilis orthologs and exhibiting putative BTS (see Fig. S1 and Data Set S1, tab 2, in the supplemental material). While E. gracilis might use VTE5 and VTE6 for both the *de novo* synthesis and salvage (38), the lack of chlorophyll and hence phytol recycling in *E. longa* implicates these enzymes only in the former function.

10.1128/mSphere.00675-20.1 FIG S1 Inferred phylogeny of phytol kinase VTE5 and phytyl-phosphate kinase VTE6. The maximum-likelihood tree was inferred with IQ-TREE using the LG+F+G4 substitution model and ultrafast bootstrapping. The UFboot support values are indicated at branches when higher than 75%. The color of the *Euglenophyceae* clade reflects possible origin in Chloroplastida (green) or Rhodophyta-derived complex algae (ochre). Download FIG S1, PDF file, 0.5 MB. Copyright © 2020 Füssy et al. 2020 Füssy et al. This content is distributed under the terms of the Creative Commons Attribution 4.0 International license.

E. gracilis is known to make tocopherols and a PhQ derivative, 5′-monohydroxyphylloquinone (OH-PhQ) (38, 42, 43). All four enzymes mediating synthesis of α-tocopherol from phytyl-PP and homogentisate were identified and are localized to its plastid (38). Interestingly, their orthologs are found in *E. longa*, all with a typical BTS or at least with the N-terminal region being highly similar to the E. gracilis counterpart (Data Set S1, tab 2), consistent with their presumed plastidial localization (Fig. 1a). Homogentisate itself is apparently made outside the plastid, as the enzyme responsible for its synthesis (4-hydroxyphenylpyruvate dioxygenase) is not found in the E. gracilis plastid proteome, and the respective proteins have a predicted mitochondrial transit peptide in both E. gracilis and *E. longa* (Data Set S1, tab 2). To test the predicted ability of *E. longa* to produce α-tocopherol, we used high-performance liquid chromatography coupled to tandem mass spectrometry (HPLC-MS/MS) to analyze extracts from this species and E. gracilis (grown in two different conditions, in light and in darkness) for comparison. Tocopherols were detected in both species (Fig. 1b), with α-tocopherol being the dominant form present in equivalent amounts in all three samples (Fig. 1c). The signals of β- and/or γ-tocopherol (indistinguishable by the method employed) and of δ-tocopherol suggest that tocopherol cyclase, and possibly also tocopherol *O*-methyltransferase, of both *Euglena* species can process substrates with or without the 3-methyl group on the benzene ring (Fig. S2).

10.1128/mSphere.00675-20.2 FIG S2 Experimental detection of tocopherols in *E. longa* and E. gracilis. (a) Overview of tocopherol biosynthesis (enzymes indicated by their EC numbers). (b to d) Extracted chromatograms of exact mass of α-tocopherol (*m*/*z* 430.3805) (inset) and recorded spectra from a particular peak in an α-tocopherol standard (b), raw lipid extracts of heterotrophic E. gracilis (c) and *E. longa* (d). (e to g) Comparison of simulation of α-tocopherol (e), β- and γ-tocopherol (f), and δ-tocopherol (g) chemical formula spectrum and experimentally gained data from the *E. longa* sample. The chemical structure of the particular tocopherol is shown as an inset. (h) Comparison of the simulated monoisotopic mass of tocopherols and high-resolution data obtained from examined euglenophyte samples. Download FIG S2, PDF file, 1.1 MB. Copyright © 2020 Füssy et al. 2020 Füssy et al. This content is distributed under the terms of the Creative Commons Attribution 4.0 International license.

The synthesis of OH-PhQ in E. gracilis is understood only partially, with only three enzymes of the pathway previously identified at the molecular level: the large multifunctional protein PHYLLO, apparently localized to the cytosol and catalyzing the first four steps leading to *o*-succinylbenzoate; MenA, catalyzing phytylation of dihydroxynaphthoate localized in the plastid; and MenG (demethylnaphthoquinone methyltransferase), possessing a typical BTS but not directly confirmed as plastidial by proteomics (38). Strikingly, *E. longa* expresses orthologs of these three E. gracilis proteins, all with the same predicted subcellular localization (Fig. 1a and Data Set S1, tab 2). As in E. gracilis, no candidates for other enzymes required for OH-PhQ synthesis could be identified by homology searches in *E. longa*. Still, OH-PhQ could be detected in this species (Fig. 1d and Fig. S3), although with a significantly lower abundance compared to E. gracilis (Fig. 1e).

10.1128/mSphere.00675-20.3 FIG S3 Experimental confirmation of 5-hydroxyphylloquinone (OH-PhQ) in *E. longa* and E. gracilis. (a) Comparison of simulation of OH-PhQ chemical formula spectrum and experimentally gained data from autotrophic E. gracilis sample. The chemical structure of OH-PhQ is shown as an inset. (b to d) Extracted chromatograms of exact mass of protonated OH-PhQ (*m*/*z* 467.35) of raw lipid extracts. The red arrow points to a peak of OH-PhQ determined by high-resolution and fragmentation patterns in autotrophic E. gracilis (b), heterotrophic E. gracilis (c), and *E. longa* (d). (e) Comparison of simulated monoisotopic mass of OH-PhQ and high-resolution data obtained from examined euglenophyte samples. Download FIG S3, PDF file, 0.5 MB. Copyright © 2020 Füssy et al. 2020 Füssy et al. This content is distributed under the terms of the Creative Commons Attribution 4.0 International license.

### The *E. longa* plastid plays a limited role in the metabolism of nitrogen-containing compounds.

Some of the apparent peculiarities of the *E. longa* plastid do not stem from the loss of photosynthesis, as they are shared with its photosynthetic relative E. gracilis. This particularly concerns plastid functions in the metabolism of nitrogen-containing compounds. Plastids are commonly involved in nitrogen assimilation due to housing nitrite reductase (44, 45), but E. gracilis (strain Z) cannot assimilate nitrate or nitrite (46, 47). Accordingly, no nitrite reductase can be identified in the transcriptome data from this species or *E. longa*. The plastids of both *Euglena* species apparently also lack the enzymes working immediately downstream of nitrite reductase, i.e., glutamine synthetase and glutamine oxoglutarate aminotransferase (the GS/GOGAT system common in plastids of other groups [48, 49]), indicating that the plastids rely on the import of organic nitrogen, similarly to what has been recently proposed for chromerids (50) and chrysophytes (20, 21).

A surprising feature of the E. gracilis plastid metabolism is the paucity of amino acid-related pathways (38). *E. longa* is even more extreme in this regard, because it lacks counterparts of the plastid-targeted serine biosynthesis enzymes. Thus, we could localize only two elements of amino acid biosynthesis pathways to the *E. longa* plastid (Fig. S4): serine/glycine hydroxymethyltransferase, whose apparent role is to provide the one-carbon moiety for formylmethionyl-tRNA synthesis required for plastidial translation; and one of the multiple isoforms of cysteine synthase A, which (as in E. gracilis) apparently relies on *O*-acetyl-l-serine synthesized outside of the plastid (see reference 38 and Data Set S1, tab 3). This is not due to incompleteness of the sequence data, as the *E. longa* transcriptome encodes enzymes for the synthesis of all 20 proteinogenic amino acids, yet their predicted localization lies outside the plastid (Data Set S1, tab 3).

10.1128/mSphere.00675-20.4 FIG S4 Plastid-linked nitrogen metabolism in *E. longa* and E. gracilis. Schematic comparison of the localization of enzymes of plastid tetrapyrrole, serine, formylmethionine, cysteine, and polyamine synthesis. For simplicity, triple arrowheads represent multiple enzymatic/transport steps in a depicted pathway. A red arrow indicates a proposed intermediate transport to support plastid precorrin synthesis in *E. longa* (details elaborated elsewhere). Abbreviations: ALAD, delta-aminolevulinic acid dehydratase; CobA, uroporphyrinogen-III C-methyltransferase; CysG, trifunctional enzyme of siroheme synthesis (see main text); CysK, cysteine synthase A; FolD, bifunctional methylenetetrahydrofolate dehydrogenase (NADP+)/cyclohydrolase; G5K, glutamate 5-kinase; GSD, glutamate semialdehyde dehydrogenase; MTA, 5′-methylthioadenosine; MTFMT, Met-tRNA formyltransferase; OAT, ornithine–oxo-acid transaminase; ODC, ornithine decarboxylase; PSAT, phosphoserine aminotransferase; PSP, phosphoserine phosphatase; (dc)SAM, (decarboxy-)*S*-adenosylmethionine; SAMD, SAM decarboxylase; SHMT, serine hydroxymethyltransferase; SpS, spermidine/spermin synthase; (f-/m-)THF, (10-formyl-/5,10-methylene-)tetrahydrofolate. Download FIG S4, PDF file, 0.4 MB. Copyright © 2020 Füssy et al. 2020 Füssy et al. This content is distributed under the terms of the Creative Commons Attribution 4.0 International license.

Amino acids also serve as precursors or nitrogen donors for the synthesis of various other compounds in plastids (51, 52). This includes tetrapyrrole synthesis, which in E. gracilis is mediated by two parallel pathways localized to the mitochondrion/cytoplasm and the plastid (53). As will be described in detail elsewhere (Z. Füssy, K. Záhonová, M. Oborník, and M. Eliáš, unpublished data), *E. longa* possesses the full mitochondrial-cytoplasmic pathway, whereas the plastidial one is restricted to its middle part potentially serving for synthesis of siroheme, but not heme and chlorophyll (Fig. S4). The spectrum of reactions related to the metabolism of other nitrogen-containing cofactors or their precursors is very limited in the plastids of both *Euglena* spp. (Data Set S1, tab 4). We identified only one such candidate in *E. longa*, vitamin B_6_ salvage catalyzed by pyridoxamine 5′-phosphate oxidase, whereas E. gracilis additionally expresses two plastid-targeted isoforms of pyridoxine 4-dehydrogenase. Enzymes of *de novo* synthesis or salvage of purines and pyrimidines are also absent from the plastid of both *Euglena* species, except for a plastidial CTP synthase isoform in E. gracilis (supported by proteomic data), which is not expressed by *E. longa*. The lack of *in situ* CTP production may reflect the presumably less extensive synthesis of RNA and/or CDP-diacylglycerol (a precursor of phospholipids) in the *E. longa* plastid. Finally, *E. longa* expresses an ortholog of spermidine synthase found in the plastid proteome of E. gracilis, but it has a modified N-terminal sequence not fitting the characteristics of a BTS, suggesting a different subcellular localization. Nevertheless, both *E. longa* and E. gracilis have another homolog of this enzyme with an obvious BTS, so polyamines may be produced in the *E. longa* plastid after all (Fig. S4).

### The *E. longa* plastid does not make fatty acids but maintains phospholipid and glycolipid synthesis.

**FIG 2:**
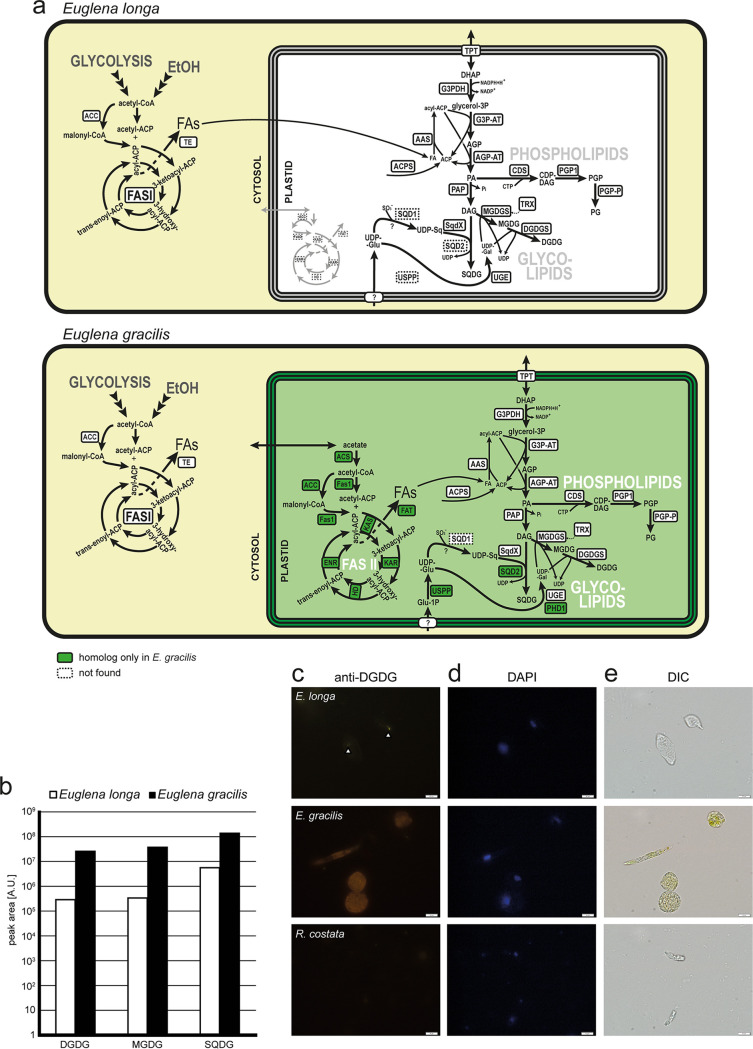
Fatty acid and lipid biosynthesis in *E. longa* and E. gracilis. (a) Schematic comparison of the localization and evolutionary origin of enzymes. Abbreviations for fatty acid (FA) synthesis: ACC, acetyl-CoA carboxylase; ACS, acetyl-CoA synthetase; ENR, enoyl-CoA reductase; EtOH, ethanol; Fas1, malonyl-CoA/acetyl-CoA:ACP transacylase; FASI, type I fatty acid synthase; FAT, fatty acyl-ACP thioesterase; HD, hydroxyacyl-ACP dehydratase; KAR, ketoacyl-ACP reductase; KAS, ketoacyl-ACP synthase; TE, fatty acid thioesterase; TRX, thioredoxin-regulated enzyme. Abbreviations for glycolipid synthesis: AAS, acyl-ACP synthase; ACPS, holo-ACP synthase; AGP-AT, acylglycerophosphate acyltransferase; G3P-AT, glycerol-3-phosphate acyltransferase; G3PDH, glycerol-3-phosphate dehydrogenase; MGDG/DGDG, mono-/digalactosyl diacylglycerol; MGDGS/DGDGS, MGDG/DGDG synthase; PAP, phosphatidic acid phosphatase; SQD1, UDP-sulfoquinovose synthase; SQD2/SQDX, sulfoquinovosyl diacylglycerol (SQDG) synthase; UGE/PHD1, UDP-glucose epimerase; USPP, UDP-sugar pyrophosphorylase. Abbreviations for phospholipid synthesis: CDS, CDP-diacylglycerol synthase; PGP1, phosphatidylglycerophosphate synthase; PGP-P, phosphatidylglycerophosphate phosphatase. (b) Semiquantitative comparison of glycolipids present in *E. longa* and autotrophically grown E. gracilis. Note the logarithmic scale of the quantification units (peak area). Peak area is shown in an arbitrary unit (A.U.) expressing the intensity of the signal of a particular lipid species, recalculated according to their respective ionization promptitude. As each lipid species has a different ionization promptitude, note that direct comparison can be done only within lipid class (for details, see reference 111). (c to e) Immunofluorescence micrographs using anti-DGDG antibody (c), DAPI (d), and differential interference contrast (DIC) (e). Autotrophic E. gracilis represents a positive control, while the aplastidic euglenozoan R. costata was used as a negative control. Bars, 10 μm.

Eukaryotes synthesize fatty acids by a single multimodular fatty acid synthase I (FASI) in the cytosol or by a multienzyme type II fatty acid synthesis complex in the plastid. E. gracilis possesses both systems (54), but *E. longa* encodes only a homolog of the cytosolic FASI enzyme (Fig. 2a; Data Set S1, tab 5). Nevertheless, *E. longa* still maintains plastid-targeted versions of acyl carrier protein (ACP) and 4′-phosphopantetheinyl transferases (or holo-ACP synthase), which are crucial for the synthesis of an active form of ACP (55). This is apparently employed by the predicted plastid-targeted homologs of acyl-ACP synthetases (presumably activating fatty acids imported into the plastid) and enzymes required for the synthesis of phosphatidic acid (PA) and its subsequent conversion to phosphatidylglycerol (PG) (Fig. 2a; Data Set S1, tab 5). Notably, *E. longa* also has a parallel, plastid-independent route of phosphatidylglycerol synthesis (Data Set S1, tab 6).

No other reactions of phospholipid synthesis or decomposition beyond PG synthesis seem to operate in the *E. longa* plastid. However, enzymes for the synthesis of galactolipids monogalactosyldiacylglycerol (MGDG) and digalactosyldiacylglycerol (DGDG) were identified, all with predicted BTSs (Fig. 2a and Data Set S1, tab 5), consistent with the plastidial localization of galactolipid synthesis in other eukaryotes (56). Moreover, both MGDG and DGDG could be detected in *E. longa* and E. gracilis by HPLC-MS/MS, although galactolipid levels were significantly lower in *E. longa* than in E. gracilis (Fig. 2b). The presence of DGDG was further confirmed by immunofluorescence using an anti-DGDG antibody, which showed DGDG to be present in small foci in the *E. longa* cells (Fig. 2c), presumably representing individual small plastids. In comparison, extensive staining was observed in E. gracilis cells consistent with plastids occupying a large portion of the cytoplasm, whereas no staining was observed in the plastid-lacking euglenid Rhabdomonas costata.

**FIG 3:**
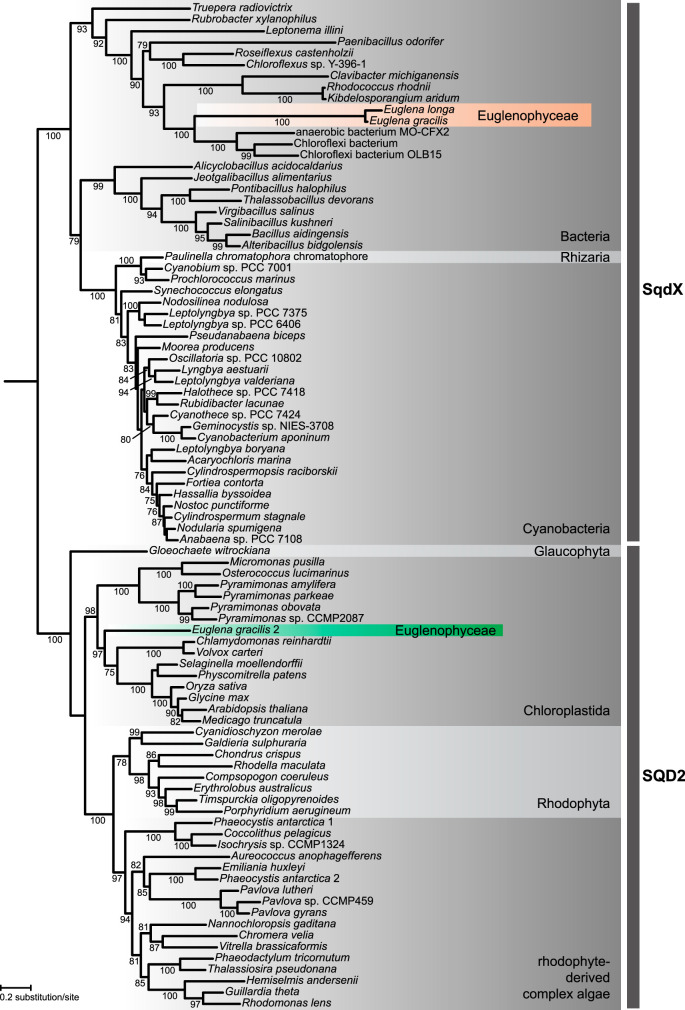
Euglenophytes have replaced the eukaryotic form of sulfoquinovosyltransferase (SQD2) with a bacterial version (SqdX). The maximum-likelihood tree was inferred with IQ-TREE using the LG+F+G4 substitution model and ultrafast bootstrapping. The UFboot support values are indicated at branches when higher than 75%. Accession numbers of sequences included in the analysis are provided in Data Set S1, tab 11, in the supplemental material.

We additionally identified another typical plastid glycolipid, sulfoquinovosyldiacylglycerol (SQDG) (57) in both *Euglena* spp. (Fig. 2b). The enzyme directly responsible for SQDG synthesis is sulfoquinovosyltransferase (Fig. 2a), but interestingly, its standard eukaryotic version (SQD2) is present only in E. gracilis, whereas both species share another isoform phylogenetically affiliated with bacterial SqdX (Fig. 3). To our knowledge, this is the first time SqdX has been found in a eukaryote. The presence of SQD2 only in E. gracilis may relate to the specific needs of its photosynthetic plastid. Indeed, E. gracilis contains much more SQDG than *E. longa* (Fig. 2b), and the profile of esterified fatty acids differs between the two species (*E. longa* lacks SQDG forms with unsaturated longer chains; Data Set S1, tab 7).

The saccharide moieties of glycolipids in *E. longa* are probably also synthesized in its plastid (Fig. 2a). *E. longa* exhibits an ortholog of the E. gracilis UDP-glucose epimerase previously identified in the plastid proteome (Fig. S5 and Data Set S1, tab 5), explaining the source of UDP-galactose for galactolipid synthesis. This seems to be an original euglenozoan enzyme recruited into the plastid (Fig. S5); interestingly, however, E. gracilis also encodes a homolog of the unique plastidial UDP-glucose epimerase (PHD1) known from plants and various algae (58). The E. gracilis PHD1 possesses a predicted BTS (Data Set S1, tab 5) and is thus also likely plastidial (albeit without proteomic support). This putative redundancy is not shared by *E. longa* (Fig. 2b) and may reflect a presumably much lower need for galactolipid synthesis. The origin of the SQDG precursor UDP-sulfoquinovose in *E. longa* remains obscure, because like E. gracilis, it lacks the conventional UDP-sulfoquinovose synthase SQD1/SqdB and probably employs an alternative, unrelated enzyme (38). UDP-glucose, i.e., the common precursor of both UDP-galactose and UDP-sulfoquinovose, may be produced directly in the plastid of E. gracilis, owing to the presence of an isoform of UDP-sugar pyrophosphorylase with a typical BTS (although absent among proteomically confirmed plastid proteins). *E. longa* lacks an ortholog of this protein as well as any other potentially plastidial enzyme of UDP-glucose synthesis (Data Set S1, tab 5), suggesting import of this metabolite from the cytosol.

10.1128/mSphere.00675-20.5 FIG S5 Inferred phylogeny of the putative plastid UDP-glucose epimerase. The maximum-likelihood tree was inferred with IQ-TREE using the best-fitting substitution model and ultrafast bootstrapping. The UFboot support values are indicated at branches when higher than 75%. The color of the *Euglenophyceae* clade reflects possible ancestral eukaryotic origin (deep pink). Download FIG S5, PDF file, 0.2 MB. Copyright © 2020 Füssy et al. 2020 Füssy et al. This content is distributed under the terms of the Creative Commons Attribution 4.0 International license.

### The *E. longa* plastid retains a linearized Calvin-Benson pathway.

**FIG 4:**
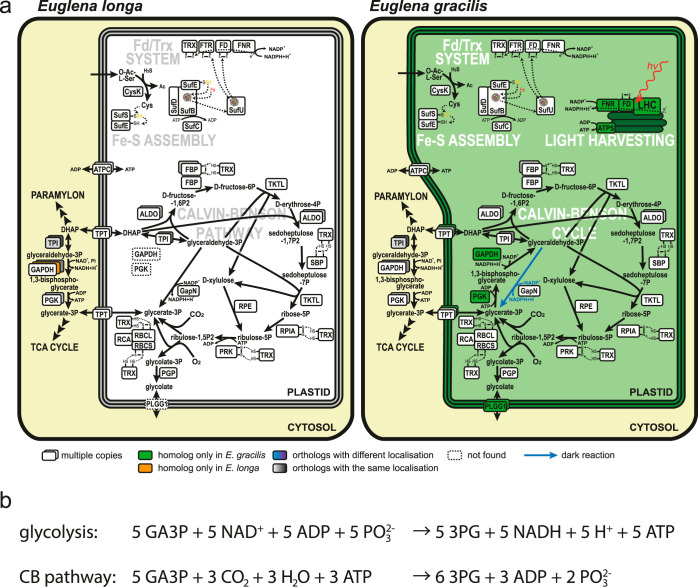
Carbon metabolism in the plastids of *E. longa* and E. gracilis. (a) The Calvin-Benson cycle (CBC) resident to this organelle is central to the plastid carbon metabolism, regulated by the ferredoxin/thioredoxin (Fd/Trx) system. Reduction of disulfide bonds by the Fd/Trx system activates several CBC enzymes (FBP, SBP, RPIA, PRK, RBCL; abbreviations explained below). FTR and FD of the Fd/Trx system require for their functioning a posttranslationally added Fe-S prosthetic group provided by the Fe-S assembly system (for details on the SUF pathway of Fe-S cluster assembly in the *E. longa* plastid, see reference 38). GapN apparently mediates the shuttling of reducing equivalent (NADPH) through the exchange of DHAP/GA3P and 3PG, reflecting the cytosolic NADPH/NADP^+^ ratio and thus the overall metabolic state of the cell. In contrast, the E. gracilis plastid is an energy-converting organelle, harvesting light into chemical energy bound as NADPH and ATP, and subsequently using this bound energy to fix CO_2_ into organic carbohydrates via the CBC. Enzyme abbreviations are color coded according to their inferred evolutionary origin; see the key. (b) Stoichiometric comparison of reactions converting glyceraldehyde 3-phosphate to 3-phosphoglycerate via glycolysis and the Calvin-Benson pathway. Abbreviations for the CB pathway: 3PG, 3-phosphoglycerate; ALDO, aldolase; DHAP, dihydroxyacetone-phosphate; FBP, fructose-1,6-bisphosphatase; GA3P, glyceraldehyde-3-phosphate; GAPDH, glyceraldehyde-3-phosphate dehydrogenase; PGK, 3-phosphoglygerate kinase; PGP, phosphoglycolate phosphatase; PLGG1, plastid glycolate/glycerate transporter; PRK, phosphoribulokinase; RBCL/RBCS, RuBisCO large/small subunit; RCA, RuBisCO activase; RPE, ribulose-5-phosphate epimerase; RPIA, ribulose-phosphate isomerase A; SBP, sedoheptulose-1,7-bisphosphatase; TCA, tricarboxylic acid; TKTL, transketolase; TPI, triose-phosphate isomerase; TPT, triose-phosphate translocator; ATPC, ADP/ATP translocase. Abbreviations for the Fd/Trx system: FD, ferredoxin; FNR, FD/NADP+ oxidoreductase; FTR, FD/TRX oxidoreductase; TRX, thioredoxin; ATPS, ATP synthase; LHC, light-harvesting complex.

The expression of both subunits of RuBisCO in *E. longa* (32) raises the question of whether the Calvin-Benson (CB) cycle (CBC) as a whole has been preserved in this organism. A putative *E. longa* plastid triose-phosphate isomerase has been described previously (59), and we additionally identified homologs with putative BTSs for nearly all remaining CBC enzymes (Data Set S1, tab 8). Phylogenetic analyses (Data Set S2) showed specific relationships of the *E. longa* proteins to the previously characterized CBC enzymes from other euglenophytes (60). However, two key CBC enzymes are apparently missing from the *E. longa* transcriptome: plastid-targeted phosphoglycerate kinase (ptPGK) and plastid-targeted glyceraldehyde-phosphate dehydrogenase (ptGAPDH). Those homologs that are present are not orthologous to the plastid-targeted isoenzymes from other euglenophytes, and all clearly lack a BTS (Data Set S1, tab 8). Hence, these are presumably cytosolic enzymes involved in glycolysis/gluconeogenesis. The lack of ptPGK and ptGAPDH in *E. longa* implies that the product of the RuBisCO carboxylase activity, 3-phosphoglycerate (3PG), cannot be converted (via 1,3-bisphosphoglycerate; 1,3-BPG) to glyceraldehyde-3-phosphate (GA3P) in the plastid (Fig. 4a).

10.1128/mSphere.00675-20.9 DATA SET S2 Phylogenetic trees in the NEWICK format. Download Data Set S2, DOCX file, 0.04 MB. Copyright © 2020 Füssy et al. 2020 Füssy et al. This content is distributed under the terms of the Creative Commons Attribution 4.0 International license.

Assuming that the reactions catalyzed by fructose bisphosphatase, phosphoribulokinase, and RuBisCO are irreversible (61), the flux through this linearized CB pathway goes from GA3P to 3PG, with a net production of six molecules of 3PG from five molecules of GA3P due to fixation of three CO_2_ molecules catalyzed by RuBisCO. Euglenophytes do not store starch in the plastid (62), and indeed, we did not find any glucose metabolism-related enzymes with a BTS in *E. longa*. Hence, GA3P cannot be produced by a glycolytic route in the *E. longa* plastid. The presence of the plastid-targeted glycerol-3-phosphate dehydrogenase (Data Set S1, tab 5) in principle allows for generation of GA3P from glycerol-3-phosphate (via dihydroxyacetone phosphate; DHAP) (Fig. 2), which could possibly come from glycerolipid turnover, but no plastidial phospholipid degradation enzymes were found in *E. longa*. Hence, the primary function of glycerol-3-phosphate dehydrogenase perhaps is to provide glycerol-3-phosphate for the plastid phospholipid and glycolipid synthesis (see above), and the *E. longa* plastid most likely imports GA3P or DHAP from the cytosol (Fig. 4a). This assumption is supported by the presence of several members of the plastid phosphate translocator (pPT) family (Fig. S6) (63), including one phylogenetically closest to a cryptophyte transporter with a preference for DHAP (64). Concerning the opposite end of the linear CB pathway, we did not identify any *E. longa* plastid-targeted enzyme that would metabolize 3PG further, suggesting that this intermediate is exported from the plastid into the cytosol, probably also by one of the pPT transporters (Fig. 4a). Obviously, the operation of the CB pathway (and of many other processes localized to the *E. longa* plastid) requires ATP supply, which is most likely mediated by ATP/ADP translocases (ATPC) orthologous to ATPC proteins identified in the E. gracilis plastid proteome (Fig. 4a and Data Set S1, tab 1).

10.1128/mSphere.00675-20.6 FIG S6 Inferred phylogeny of plastid phosphate translocators. The maximum-likelihood tree was inferred with RAxML using the best-fitting substitution model as determined by the IQ-TREE software. The UFboot support values are indicated at branches when higher than 75%. Plant translocator families with determined substrate specificity are marked. The data set was taken from reference 114. Guillardia theta and Toxoplasma gondii TPT (red) substrate specificities were determined previously to be DHAP and triose-phosphate/3-phosphoglycerate/phosphoenolpyruvate, respectively (64, 115). However, none of these translocators clusters closely with *E. longa* contigs (blue) or E. gracilis (green). Putative plastid localizations of these sequences are marked by green circles; sequences with high plastid and mitochondrial scores are marked by green and blue circles, indicating possible dual targeting. The color of the *Euglenophyceae* clade reflects the possible origin in Rhodophyta-derived complex algae (ochre). Download FIG S6, PDF file, 0.5 MB. Copyright © 2020 Füssy et al. 2020 Füssy et al. This content is distributed under the terms of the Creative Commons Attribution 4.0 International license.

RuBisCO is not only a carboxylase, but it also exhibits oxygenase activity catalyzing the production of phosphoglycolate, which is then recycled by the photorespiration pathway; this is initiated by phosphoglycolate phosphatase, yielding glycolate (65). Indeed, *E. longa* contains an ortholog of the E. gracilis plastidial phosphoglycolate phosphatase (Data Set S1, tab 8), but in contrast to E. gracilis, no homolog of the glycolate transporter PLGG1 mediating glycolate export from the plastid (66) was found in *E. longa* (Data Set S1, tab 8). Since it also lacks obvious candidates for plastid-targeted glycolate-metabolizing enzymes (glycolate oxidase, glyoxylate reductase, glycolaldehyde dehydrogenase, and glyoxylate carboligase/tartronate-semialdehyde reductase), it is unclear how glycolate is removed from the *E. longa* plastid. Possibly the amount of glycolate produced is low and can be exported by an uncharacterized PLGG1-independent route that also exists in plant plastids (67) and is sufficient for glycolate recycling in the semiparasitic plant Cuscuta campestris (68).

### The *E. longa* plastid preserves the redox regulatory system of the CB pathway.

**FIG 5:**
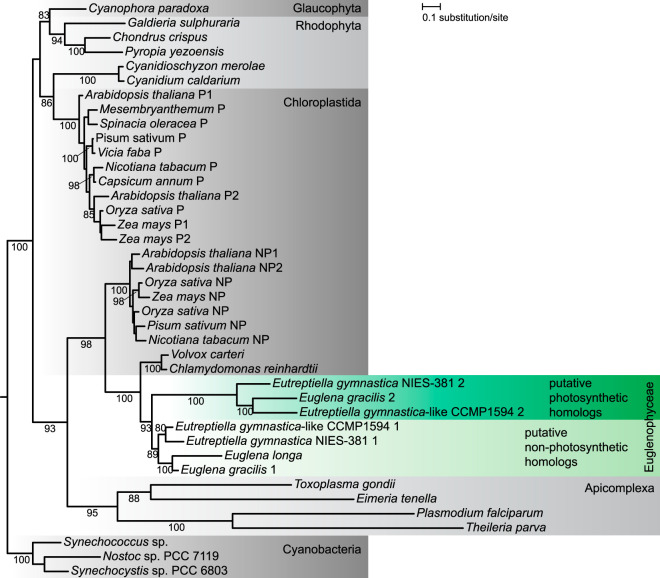
The inferred phylogeny of FNR. The maximum-likelihood tree was inferred with IQ-TREE using the LG+F+G4 substitution model and ultrafast bootstrapping. The UFboot support values are indicated at branches when higher than 75%. Euglenophyte species are shown on a green background, and their putative photosynthetic and nonphotosynthetic homologs are depicted. The two forms of plant FNR are indicated: P, photosynthetic; NP, nonphotosynthetic. Accession numbers of sequences included in the analysis are provided in Data Set S1, tab 12.

Although the photosynthetic machinery is missing from *E. longa* (37), we found homologs (with clear plastidial localization) of the typical “photosynthetic” (PetF-related) ferredoxin (Fd) and ferredoxin-NADP^+^ reductase (FNR) (Data Set S1, tab 9). These two proteins are primarily involved in passing electrons from activated photosystem I to NADP^+^. Euglenophyte FNR homologs belong to two different, yet related, clades (Fig. 5). One comprises the *E. longa* FNR and its orthologs from photosynthetic euglenophytes, whereas the second one is restricted to the photosynthetic species. Two different FNR forms also exist in plants (Fig. 5), one functioning in photosynthesis (photosystem I-dependent production of NADPH) and the other “nonphotosynthetic” one allowing electron flow in the reverse direction from NADPH to Fd (69). In analogy, we suggest that the two euglenophyte FNR forms (resulting from a gene duplication event independent of that which gave rise to the two forms in plants) functionally differ, one serving in photosynthesis and the other, also present in *E. longa*, mediating light-independent production of the reduced Fd. Multiple plastid anabolic enzymes depend on reduced Fd as an electron donor (4), but none of them seems to account for the presence of FNR and Fd in the *E. longa* plastid: glutamate synthase and nitrite reductase are missing, all identified lipid desaturases are predicted to be mitochondrion or endoplasmic reticulum (ER) targeted (Data Set S1, tab 5), and sulfite reductase, like the one previously identified in the plastid of E. gracilis (38), is NADPH dependent (Data Set S1, tab 5).

Fd also provides electrons to ferredoxin:thioredoxin reductase (FTR), mediating reduction of the protein thioredoxin (Trx). The Fd/Trx system regulates several CBC enzymes in response to the stromal redox status, whereby an excess of NADPH leads to electrons being relayed from Fd via Trx to certain disulfide bonds in the target enzymes to activate them (Fig. 4a) (70, 71). Notably, FTR and Trx homologs with an evident BTS are present in *E. longa* (Data Set S1, tab 9), and specific motifs necessary for the functioning of the Fd/Trx system are conserved in the respective *E. longa* proteins (Fig. S7). In addition, six *E. longa* CBC enzymes, fructose bisphosphatase (two of the three isoforms present), sedoheptulose bisphosphatase, phosphoribulokinase, ribose phosphate isomerase, RuBisCO large subunit (RBCL), and RuBisCO small subunit (RBCS) exhibit the conserved Trx regulatory cysteine motifs, similar to their orthologs in E. gracilis (Fig. S7 and Data Set S1, tab 10). Thus, the *E. longa* CB pathway is likely to be sensitive to the redox status in the plastid, specifically to the concentration of NADPH (Fig. 4a).

10.1128/mSphere.00675-20.7 FIG S7 Conserved cysteine motifs in ferredoxin/thioredoxin system and Calvin-Benson cycle enzymes of *E. longa* and E. gracilis. Shown are alignments of *E. longa* and E. gracilis plastid-localized homologs with reference sequences from Spinacia oleracea or Chlamydomonas reinhardtii. Catalytic residues (connected blue boxes), iron/sulfur cluster binding sites (red boxes), and redox active cysteine bonds (green boxes) are shown. Download FIG S7, PDF file, 0.5 MB. Copyright © 2020 Füssy et al. 2020 Füssy et al. This content is distributed under the terms of the Creative Commons Attribution 4.0 International license.

## DISCUSSION

The analyses described above provide evidence for the cryptic *E. longa* plastid harboring a highly unconventional combination of metabolic functions. Lacking the plastidial MEP pathway, *E. longa* joins the only recently discovered group of plastid-bearing eukaryotes with such a deficit, namely, the colorless diatom *Nitzschia* sp. strain NIES-3581 (9) and various colorless chrysophytes (20, 21). An obvious explanation for this is that the cytosolic MVA pathway is sufficient to supply precursors for all cellular isoprenoids in these organisms. In contrast, the MEP pathway in apicomplexans and related alveolates (i.e., Myzozoa) (8), and in diverse nonphotosynthetic chlorophytes (72), is essential, since the cytosolic MVA pathway has been lost in these groups (73, 74). Strikingly, our bioinformatic and biochemical evidence indicates that the *E. longa* plastid is still involved in isoprenoid metabolism, namely, the synthesis of tocopherols and phylloquinones. We thus provide independent evidence for the previous conclusion that production of phytol and its derivatives uniquely depends on the MVA pathway rather than the MEP pathway in E. gracilis (and possibly other euglenophytes) (39). As in E. gracilis, the pathway leading to OH-PhQ cannot be reconstructed in full detail in E. longa at this time (see also reference 38). Both euglenophytes studied lack homologs of the conventional enzymes of the middle part of the pathway (from *o*-succinylbenzoate to dihydroxynaphthoate) typically localized in the peroxisome (75). The respective enzyme activities were associated with the plastid envelope in E. gracilis (76), suggesting an alternative solution that may also hold for *E. longa*. The molecular identity of the putative PhQ hydroxylase (making OH-PhQ) is unknown, so its plastidial localization in E. gracilis or *E. longa* cannot be ascertained. Finally, a previously unknown step—reduction of the naphthoquinone ring—was demonstrated to be a prerequisite for the reaction catalyzed by MenG to proceed in plants and cyanobacteria (77). The respective reductase is well conserved among diverse cyanobacteria, algae, and plants (75), but we could not identify close homologs in any of the euglenophyte transcriptome assemblies, suggesting that euglenophytes employ an unknown alternative enzyme.

*E. longa* seems to be the first eukaryote with a nonphotosynthetic plastid documented to have retained the pathways for tocopherols and OH-PhQ synthesis. The presence of tocopherols in *E. longa* is not too surprising, as they are not restricted to photosynthetic tissues in plants and were also detected in nonphotosynthetic E. gracilis mutants (42, 78). As potent lipophilic antioxidants, tocopherols might be employed by *E. longa* to protect its membrane lipids against reactive oxygen species generated by mitochondria and peroxisomes. The retention of OH-PhQ synthesis in *E. longa* is more puzzling, as the best-established role of (OH-)PhQ in plants and algae is its functioning as an electron carrier within photosystem I (43, 79). PhQ was additionally proposed to serve as an electron acceptor required for the proper functioning of photosystem II (80, 81). A homolog of the respective PhQ-reducing oxidoreductase (LTO1) is present in E. gracilis (see Data Set S1, tab 2, in the supplemental material), but not in the transcriptomic data from *E. longa*. Interestingly, in plants, PhQ was also detected in the plasma membrane and has been proposed to be involved in photosynthesis-unrelated redox processes (82–84). However, the MenA and MenG enzymes in *E. longa* carry a typical BTS, suggesting that OH-PhQ in *E. longa* is involved in a hitherto uncharacterized, photosynthesis-unrelated plastid-resident process.

The absence of type II fatty acid synthesis in the *E. longa* plastid is noteworthy, yet not unprecedented, since it has been also reported for the nonphotosynthetic plastids of certain myzozoans (8) and a chrysophyte (20). Still, the *E. longa* plastid plays an active role in lipid metabolism, having retained biosynthesis of several glycerolipid types, including galactolipids and SQDG. These have previously been documented in several nonphotosynthetic algae, e.g., colorless diatoms (85, 86). On the other hand, the apicoplast (87, 88), and most likely also the relic plastid of *Helicosporidium* (based on our analysis of the respective genome data in reference 17), lacks galactolipid and SQDG synthesis completely. The reason for the differential retention of these lipids in different colorless plastids remains to be investigated further.

The truly striking feature of the *E. longa* plastid is the retention of nearly all CBC enzymes (assembling a putative linear CB pathway) and the mechanism of their redox regulation. In fact, the presence of CBC enzymes has been reported from a set of unrelated colorless algae and plants. Some of them, e.g., the dinoflagellate Crypthecodinium cohnii, the dictyochophytes Pteridomonas danica and Ciliophrys infusionum, the cryptophyte Cryptomonas paramecium, and some parasitic or mycoheterotrophic land plants, are known to carry genes that encode RuBisCO (7, 15, 89–91), but the actual complement of other CBC enzymes in these species is unknown. In contrast, transcriptomic or genomic analyses of other colorless plastid-bearing taxa, such as the dinoflagellate Pfiesteria piscicida, the chlorophyte *Helicosporidium* sp. strain ATCC 50920, the diatom *Nitzschia* sp. strain NIES-3581, and the nonphotosynthetic chrysophytes, revealed the presence of a subset of CBC enzymes, including ptPGK and ptGAPDH, but not of RuBisCO (9, 17, 21, 92). Hence, the constellation of the CBC enzymes present in the *E. longa* plastid is unique.

The CBC enzymes retained in various nonphotosynthetic eukaryotes obviously do not serve to sustain autotrophic growth due to the lack of photosynthetic production of ATP and NADPH. The incomplete CBC in *Nitzschia* was proposed to provide erythrose-4-phosphate (erythrose-4-P) for the synthesis of aromatic amino acids via the shikimate pathway (9). The data provided for the *Helicosporidium* plastid (17) offer the same explanation of the retention of several CBC enzymes. However, such rationalization cannot hold for *E. longa*, since aromatic amino acid biosynthesis in this species apparently localizes to the cytosol (Data Set S1, tab 3), thus having access to erythrose-4-P produced by the pentose phosphate pathway. In addition, *E. longa* differs from both *Nitzschia* and *Helicosporidium* by the retention of RuBisCO. A photosynthesis- and CBC-independent role of RuBisCO was described in oil formation in developing seeds of Brassica napus, where refixation of CO_2_ released during carbohydrate-to-fatty acid conversion increases carbon use efficiency (93). The absence of fatty acid synthesis in the *E. longa* plastid makes a similar function of RuBisCO in this organism unlikely.

The identification of the Fd/Trx system in the *E. longa* plastid despite the absence of photosynthesis may be key to understanding the physiological role of the linear CB pathway in *E. longa*. Another hint is provided by the discovery of a unique (nonphosphorylating) form of GAPDH, referred to as GapN, in the E. gracilis plastid (38). This enzyme uses NADP^+^ to directly oxidize GA3P to 3PG without ATP generation (94). In plants, GapN is cytosolic and involved in the shuttling of reducing equivalents from the plastid by the exchange of GA3P and 3PG between the two compartments (95). *E. longa* possesses a protein orthologous to the E. gracilis GapN with a predicted BTS (Data Set S1, tab 8), suggesting its plastidial localization. It thus appears that in *Euglena* spp., GapN mediates the shuttling of reducing equivalents in the opposite direction than in plants, i.e., from the cytosol to the plastid (Fig. 4a). In the case of *E. longa* this may be the main (if not the only) mechanism of providing NADPH for the use in the plastid, whereas E. gracilis would utilize it when photosynthetic NADPH production is shut down. At the same time, the shuttle provides a mechanism for linking the level of NADPH in the plastid with the cytosolic concentration of GA3P.

Taking all these data together, we propose that in *E. longa* (and, in specific circumstances, possibly also in E. gracilis), the plastidial NADPH/NADP^+^ ratio is directly influenced by the redox status of the cell, i.e., that it rises in an excess of reducing power that slows down the glycolytic oxidation of GA3P in the cytosol. This stimulates the linear CB pathway via the Fd/Trx system, effectively decreasing the level of GA3 by converting it to 3PG without further increasing the reducing power in the cell. This conclusion is apparent from considering the overall stoichiometries of the two alternative pathways from GA3 to 3PG (Fig. 4b). The key difference is that the CB pathway does not produce NADH that needs to be reoxidized to keep the glycolytic pathway running, since the fixed CO_2_ effectively serves as an electron acceptor. Hence, turning the CB bypass on at the expense of ATP may help the cell to keep the redox balance when reoxidation of NADH is not efficient, e.g., at hypoxic (or anoxic) conditions that simultaneously mitigate the impact of RuBisCO oxygenase activity. Indeed, euglenophytes in their natural settings are probably often exposed to oxygen shortage, and anaerobiosis in both phototrophic and heterotrophic E. gracilis has been studied to some extent (54, 96). The anaerobic heterotrophic metabolism of E. gracilis relies on fermentative degradation of paramylon, in which the mitochondrial respiratory chain uses *trans*‐2‐enoyl‐coenzyme A (CoA) as the terminal electron acceptor, eventually leading to the production of wax esters (97, 98). It is likely that *E. longa* exhibits metabolic adaptations to low oxygen levels similar to those of E. gracilis, and we propose that the plastid with the linear CB pathway might be a hitherto unrealized part of the adaptations, serving as a “redox valve” facilitating efficient metabolic flux under fluctuating oxygen concentrations. It is in fact conceivable that the same mechanism operates in E. gracilis and other photosynthetic euglenophytes when they grow heterotrophically, i.e., when the CB cycle is not engaged in production of photosynthates. Obviously, details of the euglenophyte micro- and anaerobic metabolism need to be investigated further by biochemical approaches, which are critical for testing our hypothesis and for clarifying the interplay between plastid- and mitochondrion-localized processes.

Compared to the range of forms mitochondria exhibit in diverse eukaryotes (99), plastids seem to be much more uniform. However, this is partly a reflection of our ignorance about plastid biology in most algal groups, and recent studies of various independently evolved colorless plastids document a surprising degree of diversity in terms of their metabolic capacity (100). Our analyses of the *E. longa* plastid stretch the breadth of variation among nonphotosynthetic plastids even further. The combination of pathways present (tocopherol and phylloquinone synthesis, glycolipid synthesis, and a linearized CB pathway, including RuBisCO), absent (fatty acid, amino acid, and isoprenoid precursor synthesis), and truncated (tetrapyrrole synthesis; Füssy et al., unpublished) makes the *E. longa* plastid unlike any of the previously investigated nonphotosynthetic plastids, including the apicoplast. However, further work, combining additional *in silico* analyses (aimed, e.g., at potential plastid membrane transporters mediating metabolite exchange with the cytosol) with biochemical and cytological investigations is needed to achieve a more precise idea about the protein composition of the *E. longa* plastid and a better understanding of its physiological roles.

## MATERIALS AND METHODS

### Identification and annotation of plastid-targeted proteins.

The analyses utilized the *E. longa* transcriptome assembly reported previously, with candidates for plastid-targeted proteins identified as described in reference 37, including careful manual curation of the sequences and, if needed, revision of the 5′ ends of the transcripts by targeted searches of unassembled sequencing reads. Protein models with a putative BTS were automatically annotated using InterProScan 5.21 (101). Potential plastid enzymes (references from the KEGG PATHWAY Database [https://www.genome.jp/kegg/pathway.html]) or sequences identified by literature searches and plastid proteins identified in reference 38 were searched using BLAST v.2.2.30 (against the conceptually translated proteome, the transcriptome assembly, and transcriptome sequencing [RNA-seq] reads). HMMER 3.0 (102) was used when BLAST did not yield expected candidate homologs. For comparative purposes, we used the same approach to identify plastid-targeted proteins encoded by the transcriptome assemblies from E. gracilis reported in references 98 (GenBank accession no. GDJR00000000.1) and 103 (accession no. GEFR00000000.1).

To identify orthologs of the proteins from the E. gracilis plastid proteome (38) in *E. longa*, reciprocal BLAST searches were used. Briefly, E. gracilis proteins identified in its plastid proteome were used as queries in tBLASTn searches in the *E. longa* transcriptome with an E-value cutoff of 0.1. Each respective best BLAST hit from *E. longa* was then used as a query to search the whole E. gracilis transcriptomic database, and it was classified as an ortholog if it retrieved the original E. gracilis query as a first hit. Results are summarized in Data Set S1, tab 1, in the supplemental material.

For MenA cDNA resequencing, mRNA was extracted using the TRI reagent and the Dynabeads mRNA purification kit (both from Thermo Fisher Scientific, Waltham, MA, USA). Reverse transcription was performed with random hexamers and StrataScript III reverse transcriptase (Thermo Fisher Scientific). The target was amplified using forward 5′-GGTGCTGTTCTGCTCTCACT-3′ and reverse 5′-CAGTGGGGATCAGAGATGCG-3′ primers and Q5 high-fidelity DNA polymerase in a standard buffer solution (New England Biolabs, Ipswich, USA). Amplicons were purified on MinElute PCR purification columns (Qiagen, Hilden, Germany) and sequenced at the GATC sequencing facility (Konstanz, Germany).

### Phylogenetic analyses.

Homologs of target proteins were identified using BLAST v.2.2.30 searches in the nonredundant protein sequence database at NCBI (www.ncbi.nlm.nih.gov) and among protein models of selected organisms from JGI (genome.jgi.doe.gov) and MMETSP (imicrobe.us/#/projects/104) (104). Sequences were aligned using the MAFFT v7.407 tool with the L-INS-I setting (105), and poorly aligned positions were eliminated using trimAl v1.4.rev22 with “-automated1” trimming (106). For presentation purposes, alignments were processed using CHROMA (107). Maximum-likelihood trees were inferred using the LG+F+G4 model of IQ-TREE v1.6.9 (108), employing the strategy of rapid bootstrapping followed by a “thorough” likelihood search with 1,000 bootstrap replicates. The list of species and the numbers of sequences and amino acid positions are presented in Data Set S1, tabs 11 to 22, for each phylogenetic tree.

### Culture conditions.

Euglena gracilis strain Z (“autotrophic” conditions) was cultivated statically under constant illumination at 26°C in Cramer-Myers medium with ethanol (0.8% [vol/vol]) as a carbon source (109). *E. longa* strain CCAP 1204-17a (a gift from Wolfgang Hachtel, Bonn, Germany) and heterotrophic E. gracilis strain Z were cultivated as described above, but without illumination. *Rhabdomonas costata* strain PANT2 (a gift from Vladimír Hampl, Charles University, Prague, Czech Republic) was isolated from a freshwater body in Pantanal (Brazil) and grown with an uncharacterized mixture of bacteria in Sonneborn’s *Paramecium* medium (pH 7.4) (110) at room temperature.

### Mass spectrometry of structural lipids and terpenoids.

Lipid extracts from *E. longa* and autotrophically grown E. gracilis cellular pellets (four biological samples of different culture ages) were obtained with procedures described in reference 111. Briefly, approximately 10 mg (wet weight) of both harvested cultures were homogenized by using a TissueLyser LT mill (Qiagen) and extraction was performed using a chloroform-methanol solution (2:1 ratio) following the previously described method (112). Aliquots from each sample were analyzed using an HPLC MS system powered by a linear ion trap LTQ-XL mass spectrometer (Thermo Fisher Scientific). The settings of the system were set according to the previously published methodology (111). Data were acquired and processed using Xcalibur software version 2.1 (Thermo Fisher Scientific). Particular compounds were determined based on an earlier publication (111). Terpenoids were extracted from an autotrophic and heterotrophic culture of E. gracilis, and a culture of *E. longa* of the same age in three replicates. The same extraction protocol as for lipid analysis was used. Sample aliquots were injected into the high-resolution mass spectrometry system powered by Orbitrap Q-Exactive Plus with a Dionex Ultimate 3000 XRS pump and Dionex Ultimate 3000 XRS Open autosampler (both from Thermo Fisher Scientific), and the settings described in reference 111 were used. Data were acquired and processed using Xcalibur software version 2.1. Identification of OH-PhQ was done by considering the *m/z* value, fragmentation pattern, and high-resolution data. Tocopherols (α, β/γ, and δ) were determined by the same characteristics as those used for OH-PhQ, and results were then compared with commercially purchased standards (Sigma-Aldrich, St. Louis, MO, USA).

### Immunofluorescence assay.

Immunofluorescence was performed as previously described (113). Briefly, cells were fixed in 4% paraformaldehyde for 30 min, permeabilized for 10 min on ice with 0.1% Igepal CA-630 (Sigma-Aldrich) in PHEM buffer (pH 6.9) [60 mM piperazine-*N*,*N*′-bis(2-ethanesulfonic acid) (PIPES), 25 mM HEPES, 10 mM EGTA, 2 mM MgCl_2_], and background was masked with 3% bovine serum albumin (BSA) in PHEM buffer. DGDG was detected using a polyclonal rabbit anti-DGDG antibody (1:25), a kind gift from Cyrille Y. Botté (University of Grenoble I, Grenoble, France), followed by incubation with a secondary Cy3-labeled polyclonal goat anti-rabbit antibody (AP132C, 1:800, Merck Millipore, Burlington, MA, USA). Cells were mounted on slides using Fluoroshield with 4′,6′-diamidino-2-phenylindole (DAPI) mounting medium (Sigma-Aldrich) and observed with an Olympus BX53 microscope (Olympus, Tokyo, Japan).

### Data availability.

The MenA cDNA sequence is deposited in GenBank (accession no. MK484704).
