# Supplementary figures and images for: The Cryptic Plastid of Euglena longa Defines a New Type of Nonphotosynthetic Plastid Organelle
Source: mSphere. 2020 Oct 21;5(5):e00675-20. doi: 10.1128/mSphere.00675-20 (PMC7580956; doi:10.1128/mSphere.00675-20)

## VTE5

0.2 substitution/site

## VTE6

0.2 substitution/site

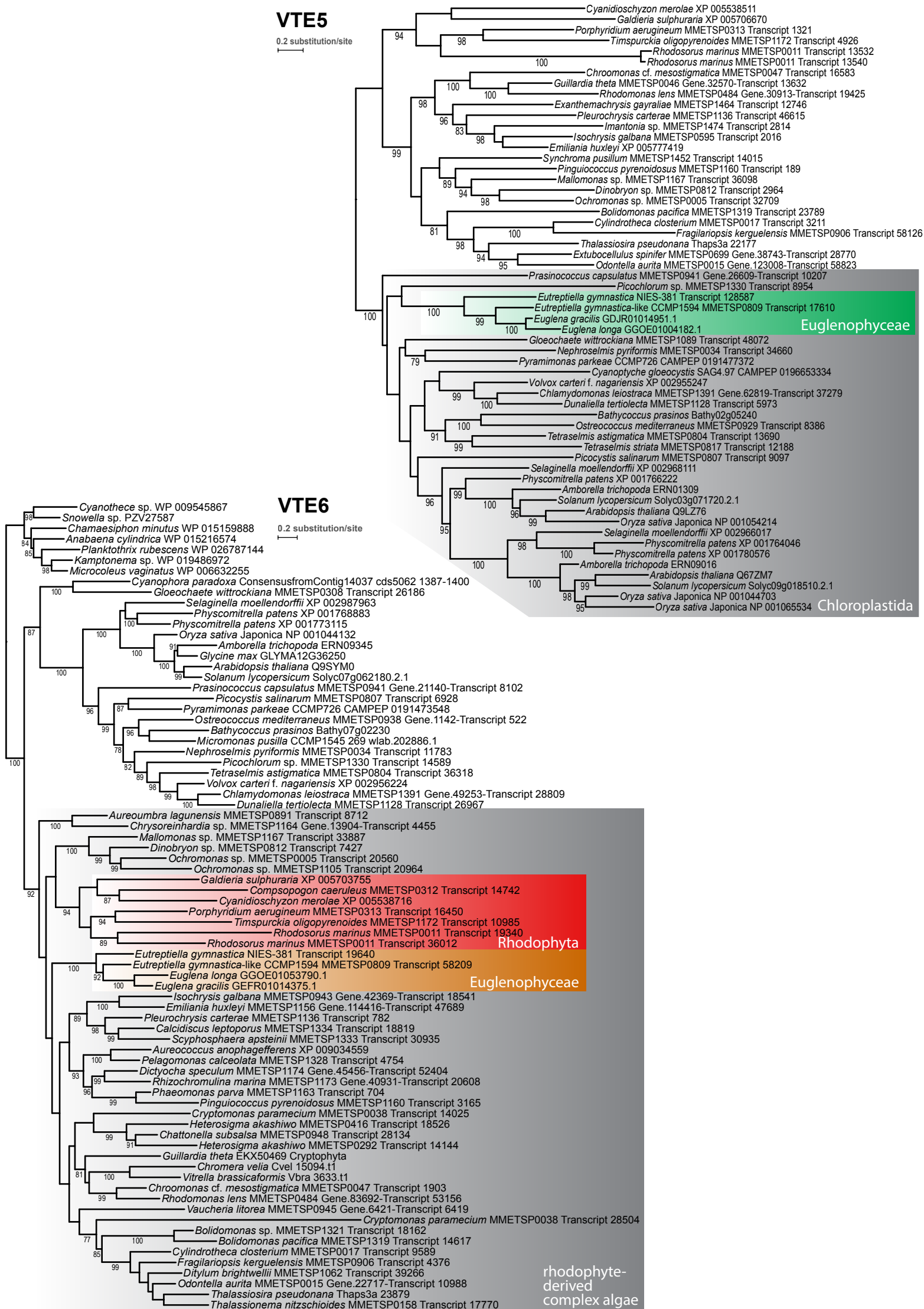

Supplement: FIG S1 [file mSphere.00675-20-sf001.pdf]

# UDP-glucose epimerase

0.1 substitutions/site

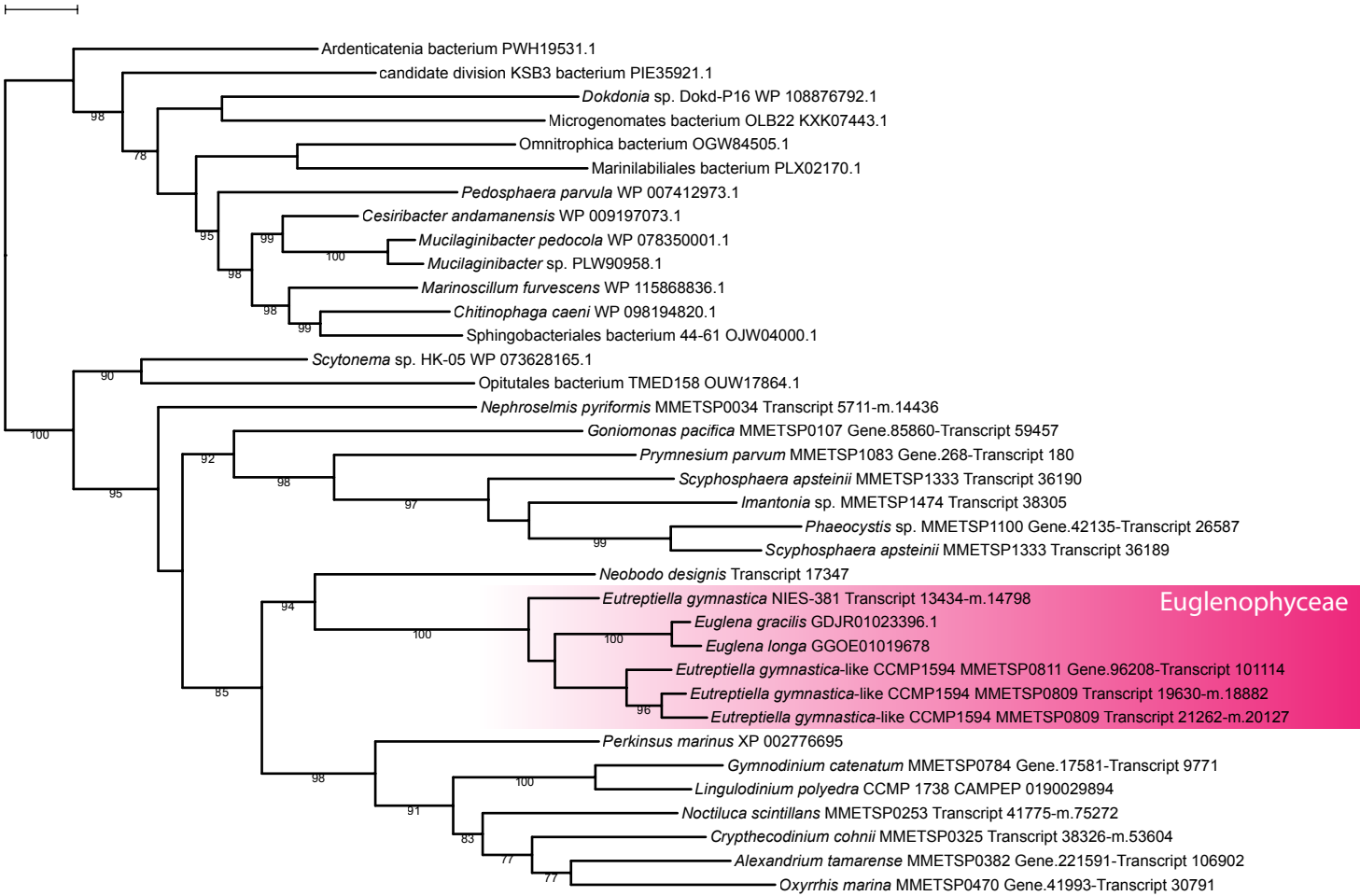

Supplement: FIG S5 [file mSphere.00675-20-sf005.pdf]

## 1 substitution/site

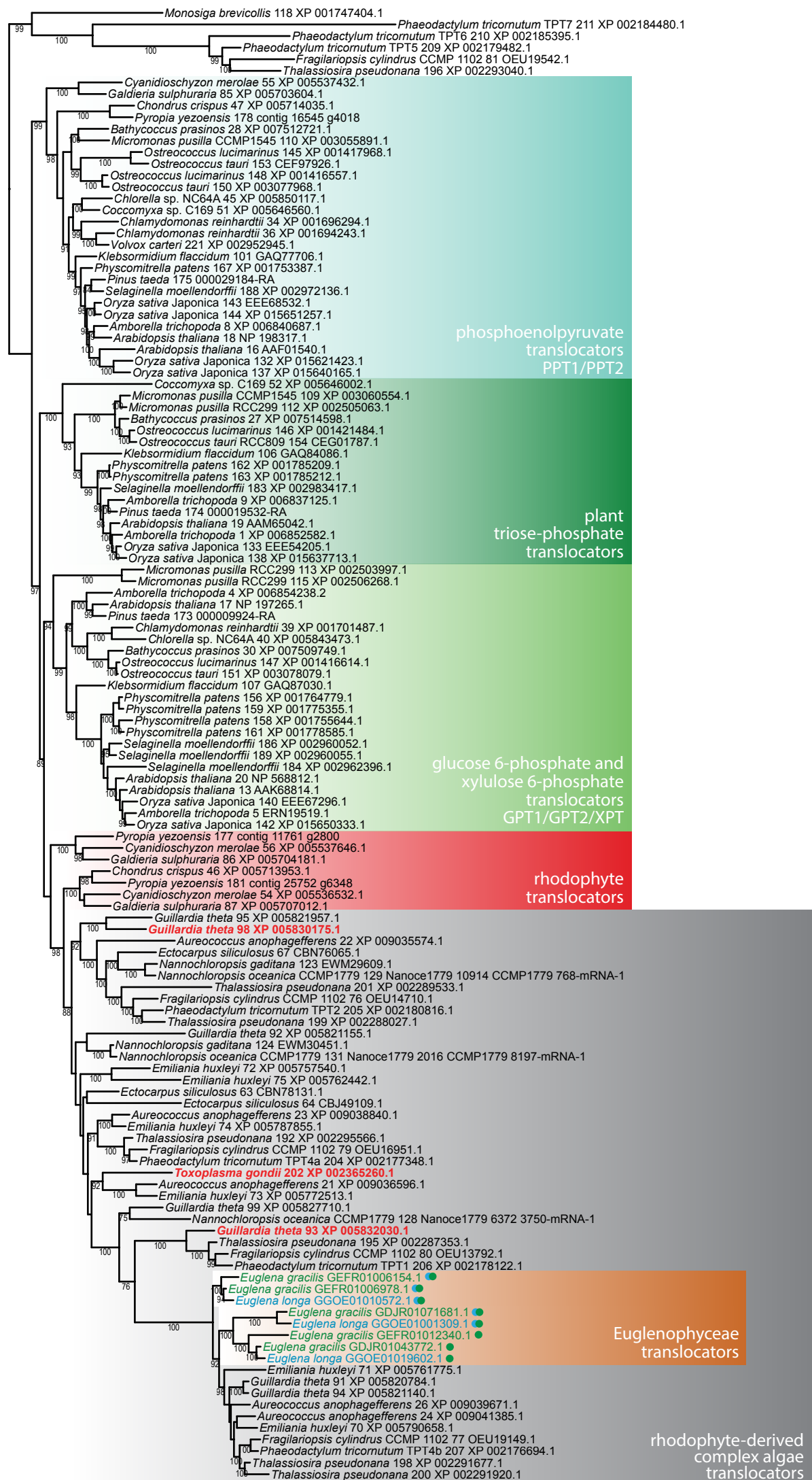

Supplement: FIG S6 [file mSphere.00675-20-sf006.pdf]
