# Supplementary material for: The Cryptic Plastid of Euglena longa Defines a New Type of Nonphotosynthetic Plastid Organelle
Source: mSphere. 2020 Oct 21;5(5):e00675-20. doi: 10.1128/mSphere.00675-20 (PMC7580956; doi:10.1128/mSphere.00675-20)
Supplement: FIG S2 [file mSphere.00675-20-sf002.pdf]

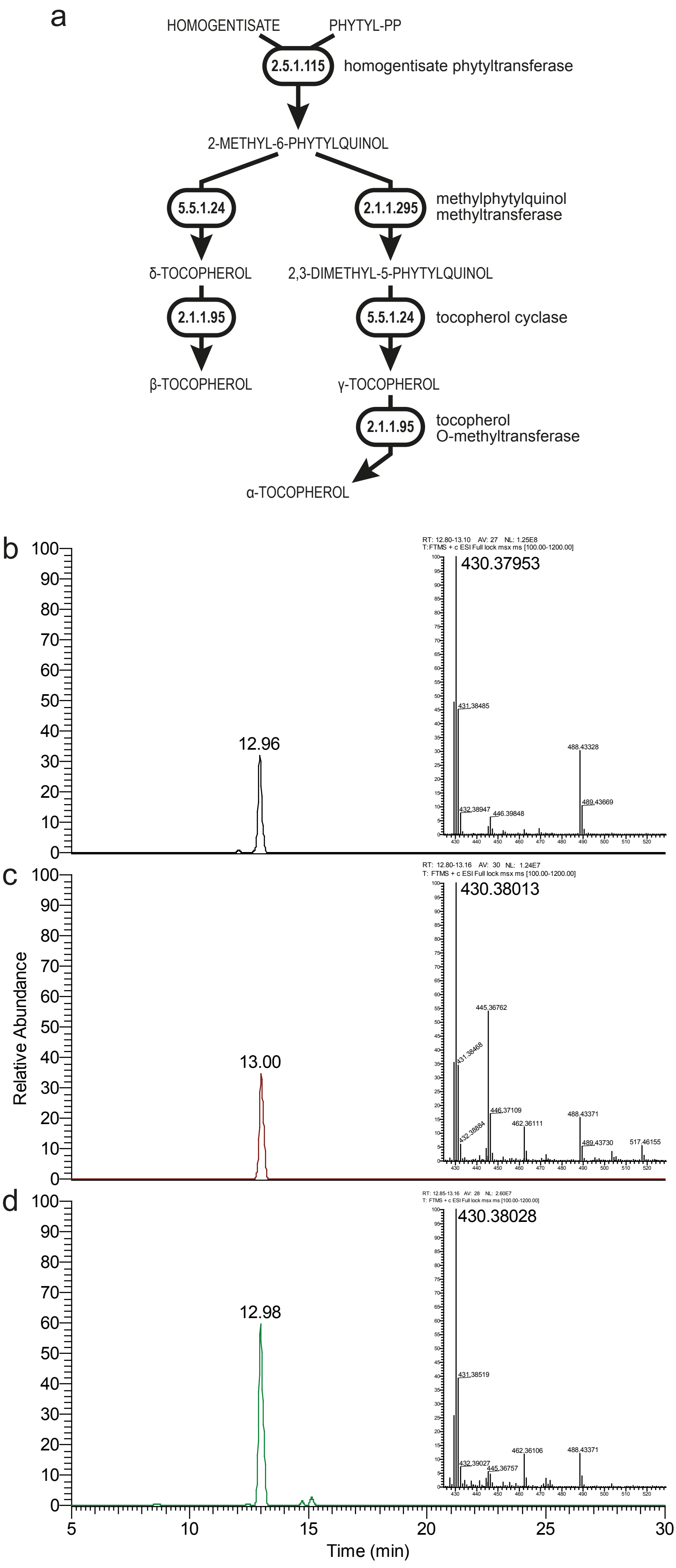

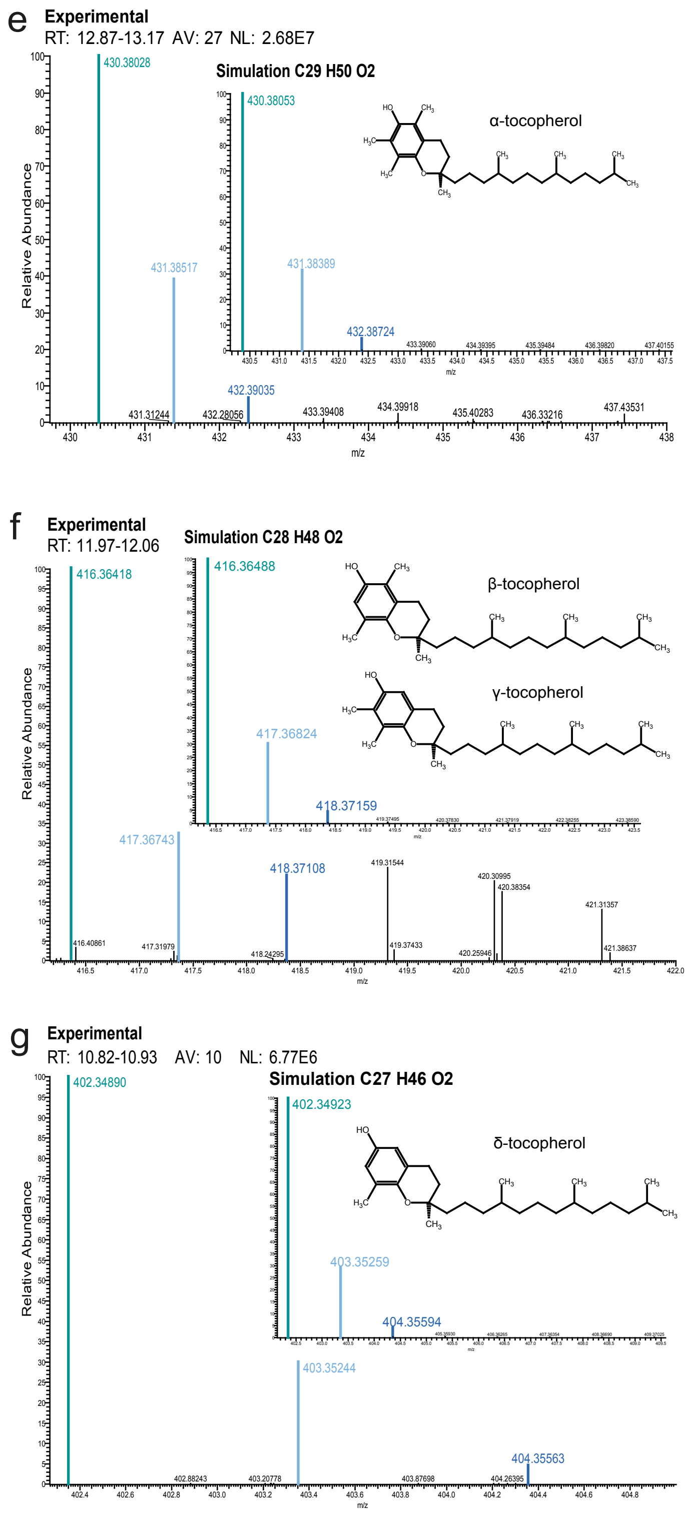

h

		α-tocopherol	β-/γ-tocopherol	δ-tocopherol
**simulation**		430.38052	416.36488	402.34923
**standards**	**[M]+[Da]**	430.37947	416.36443	402.34897
***E. gracilis* heterotrophic**		430.38343	416.36440	402.35766
E. longa		430.38053	416.36418	402.34890
**standards**		-0.00105	-0.00045	-0.00026
***E. gracilis* heterotrophic**	**Δ [M]+[Da]**	0.00396	-0.00048	0.00843
E. longa		-0.00290	-0.00070	-0.00033
