# Supplementary material for: The Cryptic Plastid of Euglena longa Defines a New Type of Nonphotosynthetic Plastid Organelle
Source: mSphere. 2020 Oct 21;5(5):e00675-20. doi: 10.1128/mSphere.00675-20 (PMC7580956; doi:10.1128/mSphere.00675-20)
Supplement: FIG S3 [file mSphere.00675-20-sf003.pdf]

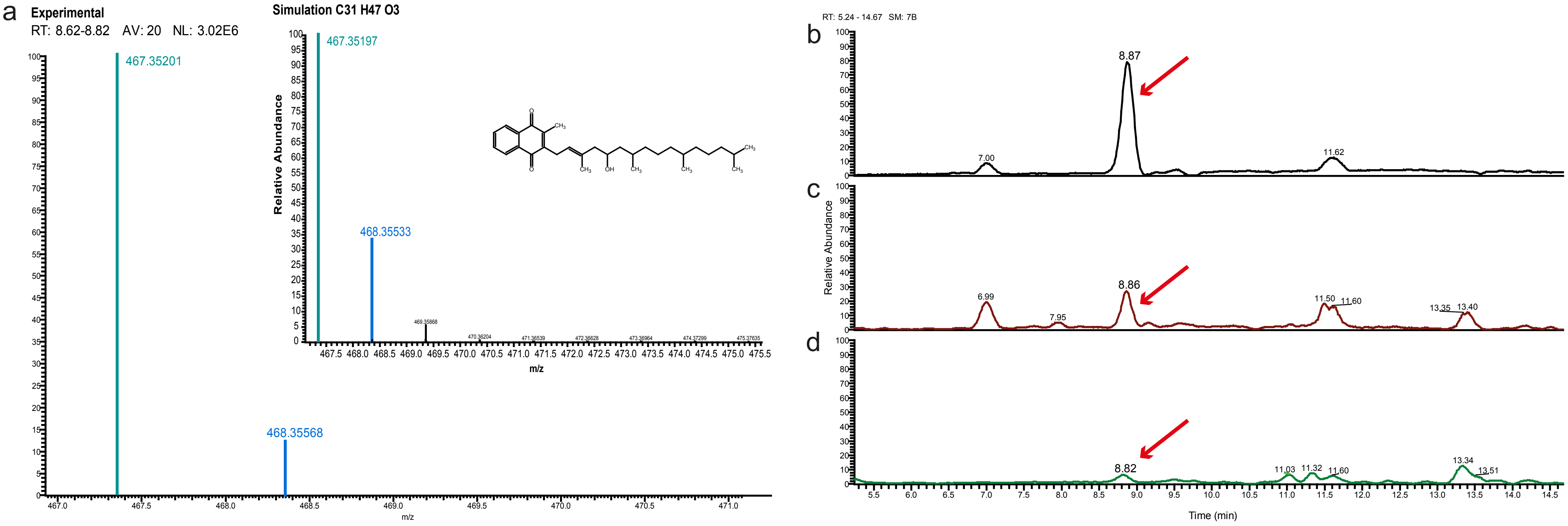

e

5 - hydroxyphylloquinone	$[M+H]^+$ [Da]	Δ $[M+H]^+$[Da]
simulation	467.35197	
E. gracilis autotrophic	467.35191	0.00006
E. gracilis heterotrophic	467.35196	0.00001
E. longa	467.35202	-0.00005
