# Supplementary material for: The Cryptic Plastid of Euglena longa Defines a New Type of Nonphotosynthetic Plastid Organelle
Source: mSphere. 2020 Oct 21;5(5):e00675-20. doi: 10.1128/mSphere.00675-20 (PMC7580956; doi:10.1128/mSphere.00675-20)
Supplement: FIG S4 [file mSphere.00675-20-sf004.pdf]

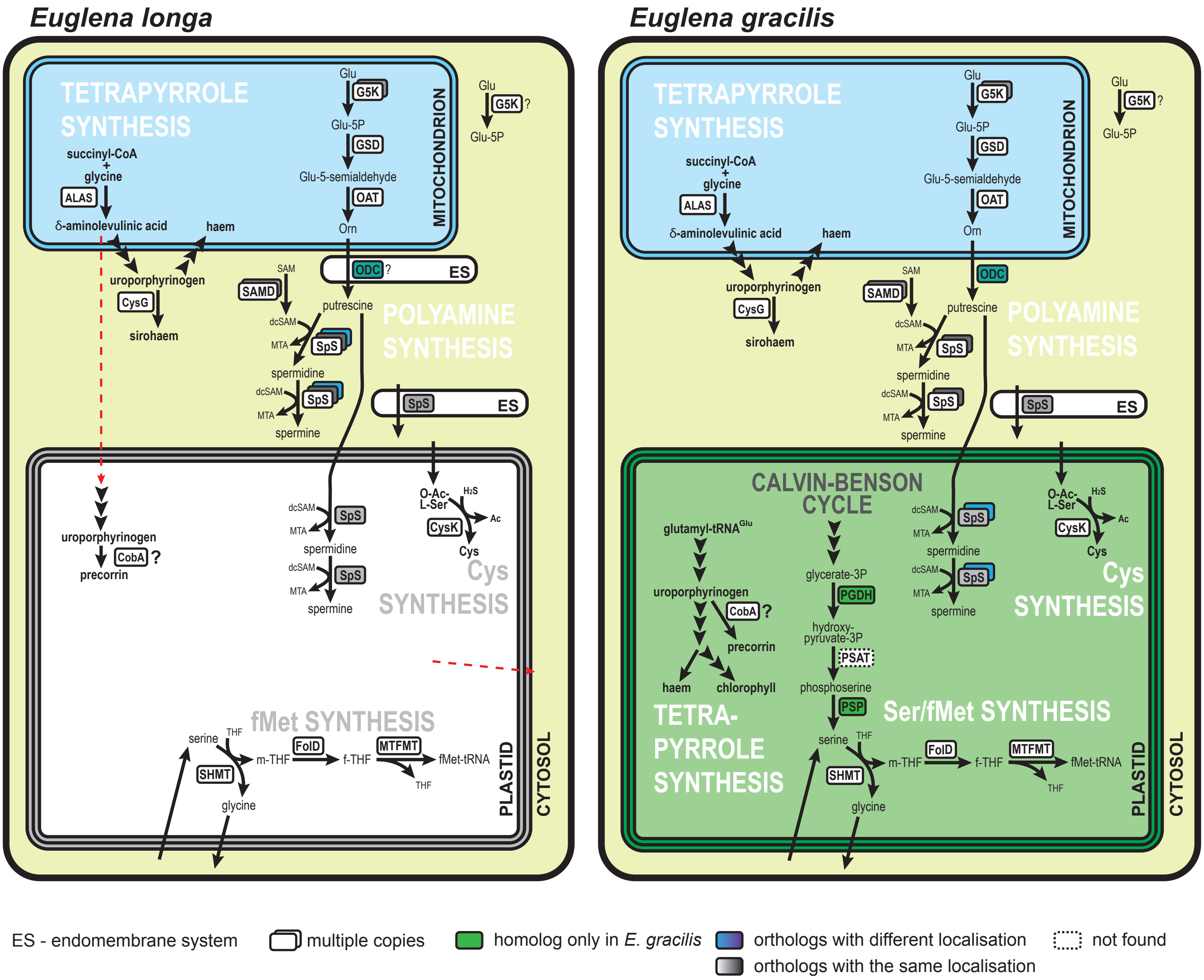

Euglena longa
Euglena gracilis
TETRAPYRROLE SYNTHESIS
succinyl-CoA + glycine
ALAS
δ-aminolevulinic acid
haem
uroporphyrinogen
CysG
sirohaem
Glu
G5K
Glu-5P
GSD
Glu-5-semialdehyde
OAT
Orn
MITOCHONDRION
ODC
ES
SAM
SAMD
putrescine
POLYAMINE SYNTHESIS
dcSAM
MTA
SpS
spermidine
spermine
CobA
precorrin
O-Ac-L-Ser
H2S
Ac
CysK
Cys
Cys SYNTHESIS
fMet SYNTHESIS
serine
THF
SHMT
m-THF
FolD
f-THF
MTFMT
fMet-tRNA
glycine
PLASTID
CYTOSOL
CALVIN-BENSON CYCLE
glutamyl-tRNA Glu
glycerate-3P
PGDH
hydroxy-pyruvate-3P
PSAT
phosphoserine
PSP
chlorophyll
TETRA-PYRROLE SYNTHESIS
Ser/fMet SYNTHESIS
ES - endomembrane system
multiple copies
homolog only in E. gracilis
orthologs with different localisation
orthologs with the same localisation
not found
