# Supplementary material for: The Cryptic Plastid of Euglena longa Defines a New Type of Nonphotosynthetic Plastid Organelle
Source: mSphere. 2020 Oct 21;5(5):e00675-20. doi: 10.1128/mSphere.00675-20 (PMC7580956; doi:10.1128/mSphere.00675-20)
Supplement: DATA SET S2 [file mSphere.00675-20-sd002.docx]

**Supplementary data to the paper:**

**The cryptic plastid of *Euglena longa* defines a new type of non-photosynthetic plastid organelles**

Zoltán Füssy^1,2,3^, Kristína Záhonová^1,2,4^, Aleš Tomčala^1,*^, Juraj Krajčovič^5^, Vyacheslav Yurchenko^4^, Miroslav Oborník^1,3^, Marek Eliáš^4,#^

^1^ Institute of Parasitology, Biology Centre ASCR, České Budějovice, Czech Republic

^2^ Faculty of Science, Charles University, BIOCEV, Vestec, Czech Republic

^3^ University of South Bohemia, Faculty of Science, České Budějovice, Czech Republic

^4^ Life Science Research Centre, Department of Biology and Ecology and Institute of Environmental Technologies, Faculty of Science, University of Ostrava, Ostrava, Czech Republic

^5^ Department of Biology, Faculty of Natural Sciences, University of ss. Cyril and Methodius in Trnava, Trnava, Slovakia

Running Head: Metabolic roles of the *Euglena longa* cryptic plastid

#Address correspondence to Marek Eliáš, marek.elias@osu.cz.

*Present address: University of South Bohemia, Faculty of Fisheries and Protection of Waters, CENAKVA, České Budějovice, Czech Republic

Zoltán Füssy and Kristína Záhonová contributed equally to this work. Author order was determined on the basis of seniority.

**Fructose bisphosphate aldolase, dataset listed in Data Set S1, Tab 13:**

((Drosophila melanogaster1:0.12675,Drosophila melanogaster2:0.34855):0.28522,(((Lotharella sp. CCMP6222:0.51769,((Chlorella variabilis1:0.23372,(((((((Arabidopsis thaliana2:0.00862,Arabidopsis thaliana3:0.03349)100:0.03534,Arabidopsis thaliana7:0.05720)97:0.02781,((Oryza sativa Japonica2:0.13246,Oryza sativa Japonica 6:0.03642)100:0.04124,Oryza sativa Japonica4:0.12334)91:0.01251)100:0.03893,(Selaginella moellendorffii2:0.10974,Selaginella moellendorffii3:0.23528)82:0.02889)81:0.02775,((Arabidopsis thaliana4:0.12856,Arabidopsis thaliana9:0.01371)100:0.09255,Oryza sativa Japonica3:0.06824)98:0.03459)91:0.03104,(Physcomitrella patens2:0.07516,Physcomitrella patens4:0.04547)100:0.18773)93:0.04574,Physcomitrella patens5:0.12477)97:0.05448)51:0.03765,Coccomyxa subellipsoidea3:0.20150)67:0.02054)47:0.02450,((((Dictyostelium discoideum:0.28215,((Guillardia theta1:0.11331,Rhodomonas salina2:0.25097)100:0.15052,Cyanidioschyzon merolae1:0.24437)87:0.06092)63:0.02869,(Galdieria sulphuraria2:0.14243,Porphyridium aerugineum2:0.22074)100:0.10060)37:0.02192,(Cryptomonas paramecium1:0.11987,(Guillardia theta2:0.45057,Rhodomonas salina1:0.12742)98:0.07326)100:0.16735)68:0.04526,((((((Neospora caninum1:0.01930,Toxoplasma gondii1:0.02540)100:0.06336,(Neospora caninum2:0.07014,Toxoplasma gondii2:0.00270)99:0.04110)100:0.15122,((Perkinsus marinus1:0.13447,Perkinsus marinus2:0.07994)69:0.03943,Perkinsus marinus3:0.10738)100:0.12979)57:0.04532,((Paramecium tetraurelia1:0.01505,Paramecium tetraurelia2:0.03012)100:0.10633,Tetrahymena thermophila:0.17386)100:0.25161)65:0.04456,(((((Bathycoccus prasinos1:0.32307,(((((Chlamydomonas reinhardtii2:0.02206,Volvox carteri f. nagariensis1:0.02769)100:0.06080,Dunaliella tertiolecta:0.11421)100:0.02248,Polytomella parva:0.17973)99:0.02442,Coccomyxa subellipsoidea1:0.11107)52:0.02989,Chlorella variabilis2:0.10099)100:0.02185)100:0.06239,(((Arabidopsis thaliana1:0.05963,Oryza sativa Japonica1:0.04591)99:0.05876,((((Arabidopsis thaliana5:0.00000,Arabidopsis thaliana8:0.00000)100:0.02248,Arabidopsis thaliana6:0.03123)100:0.03730,Oryza sativa Japonica5:0.04723)100:0.04621,Selaginella moellendorffii1:0.09996)97:0.02541)86:0.01798,((Physcomitrella patens1:0.01807,Physcomitrella patens8:0.01408)100:0.04537,(Physcomitrella patens6:0.02945,Physcomitrella patens7:0.01123)100:0.03545)94:0.02016)98:0.06793)100:0.26753,((Cryptomonas paramecium2:0.19993,((Prochlorococcus marinus:0.34895,Ectocarpus siliculosus2:0.16076)96:0.06276,((Chondrus crispus1:0.09026,(Porphyridium aerugineum1:0.05400,Rhodella maculata1:0.16249)100:0.05906)100:0.04030,(Cyanidioschyzon merolae2:0.21968,Galdieria sulphuraria1:0.09328)97:0.03033)99:0.04922)98:0.02174)96:0.07516,Phaeodactylum tricornutum:0.46689)98:0.05001)89:0.05467,((((((((Chlamydomonas reinhardtii1:0.03675,Volvox carteri f. nagariensis2:0.04117)100:0.04208,Dunaliella tertiolecta1:0.08118)99:0.04019,Coccomyxa subellipsoidea2:0.13049)98:0.04690,Nannochloropsis gaditana:0.21287)45:0.02373,Physcomitrella patens3:0.13292)100:0.14302,Chondrus crispus2:0.30903)100:0.17830,(((Chlamydomonas reinhardtii:0.14697,Volvox carteri f. nagariensis3:0.05660)100:0.35719,Ectocarpus siliculosus3:0.62354)34:0.04741,Chondrus crispus3:0.66344)95:0.09267)47:0.05311,(Euglena gracilis3 PT:0.03990,Euglena longa1 PT:0.02703)100:0.32738)55:0.05155)56:0.03886,(Cyanoptyche gloeocystis:0.27354,((Cyanoptyche gloeocystis2:0.12511,Cyanoptyche gloeocystis3:0.12058)97:0.05853,Gloeochaete wittrockiana2:0.21291)95:0.05016)97:0.04898)41:0.01527)62:0.04948,(Micromonas pusilla2:0.27125,(Ostreococcus tauri1:0.42830,(((Diplonema papillatum:0.29419,(Leptomonas pyrrhocoris:0.09482,Trypanosoma brucei:0.09806)100:0.21065)100:0.38482,Naegleria gruberi:0.49149)91:0.03315,(Nitrobacter hamburgensis:0.39365,Cyanothece sp. PCC 7425:0.47925)100:0.22485)92:0.07354)78:0.04153)87:0.08428)52:0.01731)42:0.01591)70:0.04962,(Lotharella sp. CCMP6223:0.41771,(Gloeochaete wittrockiana:0.30837,(((Lotharella sp. CCMP6221:0.14047,(((Bathycoccus prasinos2:0.09508,Ostreococcus tauri2:0.10580)100:0.07718,Micromonas pusilla1:0.11592)98:0.07090,((Pyramimonas amyliferam:0.16575,Rhodella maculata2:0.50181)96:0.03964,((Euglena gracilis1 PT:0.02104,Euglena longa3 PT:0.07741)100:0.08905,(Eutreptiella gymnastica-like CCMP1594:0.09637,Eutreptiella gymnastica NIES-381:0.05219)99:0.04858)88:0.05169)92:0.03397)100:0.10271)100:0.07412,(Guillardia theta3:0.15680,Ectocarpus siliculosus:0.15417)100:0.10262)100:0.41713,(Euglena gracilis2:0.06640,Euglena longa2:0.10176)100:0.21233)100:0.37878)92:0.05354)52:0.04675):0.01501)100;

**Fructose bisphosphatase, dataset listed in Data Set S1, Tab 14:**

((((((((((((((((((('Eutreptiella gymnastica NIES-381 2':0.15887699999999993,'Eutreptiella gymnastica NIES-381 3':0.10662800000000017)99:0.042946999999999846,'Eutreptiella gymnastica NIES-381 1':0.12924300000000022)95:0.049059000000000186,'Eutreptiella gymnastica-like CCMP1594 1':0.04077900000000012)96:0.04304100000000011,'Euglena longa 7 PT':0.019550999999999874)65:0.006728999999999985,'Euglena gracilis 1 PT':0.018918000000000212)96:0.07441999999999993,('Euglena gracilis 3 PT':0.025993000000000155,'Euglena longa 3 PT':0.0657359999999998)100:0.11893500000000001)99:0.1182120000000002,'Emiliania huxleyi2':0.411127)99:0.06207100000000043,('Phaeodactylum tricornutum1':0.09981600000000013,'Phaeodactylum tricornutum4':0.0826570000000002)100:0.19954300000000025)99:0.0630799999999998,((('Guillardia theta':0.042222000000000204,'Rhodomonas salina2':0.06005899999999986)82:0.04644199999999987,'Cryptomonas paramecium':0.09594700000000023)100:0.15610800000000014,'Emiliania huxleyi3':0.16472599999999993)100:0.06627699999999992)99:0.05322099999999974,(('Phaeodactylum tricornutum2':0.14657799999999988,'Thalassiosira pseudonana2':0.1809210000000001)100:0.3103030000000002,'Ectocarpus siliculosus2':0.6496560000000002)100:0.38211799999999974)80:0.06268700000000038,((('Phaeodactylum tricornutum5':0.32893300000000014,'Thalassiosira pseudonana1':0.3466070000000001)100:0.43864400000000003,'Ectocarpus siliculosus1':0.14325300000000007)69:0.005465000000000053,'Aureococcus anophagefferens1':0.2012900000000002)65:0.02129700000000012)66:0.040765999999999636,'Lotharella sp. CCMP6223':0.28142699999999987)60:0.03072800000000031,(((('Porphyridium aerugineum3':0.305971,'Porphyridium aerugineum1':0.14819300000000002)94:0.0709850000000003,'Chondrus crispus2':0.06537800000000038)82:0.014049999999999674,('Rhodella maculata1':0.02111900000000011,'Rhodella maculata2':0.0032840000000002867)100:0.16647699999999999)85:0.03274599999999994,(('Galdieria sulphuraria':0.31458200000000014,'Galdieria sulphuraria2':0.1218309999999998)96:0.028289999999999704,'Cyanidioschyzon merolae':0.3561599999999996)96:0.08015000000000017)96:0.08085999999999993)78:0.105715,((((((((('Chlamydomonas reinhardtii':0.017441999999999958,'Volvox carteri f. nagariensis':0.026727000000000167)100:0.034191000000000304,'Polytomella parva':0.11968799999999957)100:0.03473999999999977,'Dunaliella tertiolecta2':0.1565669999999999)100:0.04584400000000022,'Chlorella variabilis2':0.29544599999999965)97:0.03007900000000019,'Coccomyxa subellipsoidea3':0.12199900000000019)96:0.04178499999999952,'Pyramimonas parkeae':0.367289)17:0.000002,((('Bathycoccus prasinos':0.21864099999999986,'Micromonas pusilla3':0.19259799999999982)100:0.42585300000000004,'Micromonas pusilla':0.12205399999999988)66:0.040863000000000316,('Bathycoccus prasinos2':0.16168499999999986,'Ostreococcus tauri':0.040134000000000114)100:0.08139200000000013)72:0.03501099999999946)84:0.04786900000000038,((('Physcomitrella patens2':0.023608000000000295,'Physcomitrella patens3':0.01407500000000006)100:0.05701299999999998,'Selaginella moellendorffii3':0.07462900000000028)76:0.02430500000000002,('Arabidopsis thaliana2':0.029383999999999855,'Oryza sativa2':0.077909)78:0.02223200000000025)99:0.050060000000000215)99:0.09970799999999969,((('Coccomyxa subellipsoidea2':0.20696599999999998,'Dunaliella tertiolecta':0.3928449999999999)41:0.07094000000000023,'Porphyridium aerugineum4':0.5125489999999999)99:0.14308500000000013,(('Euglena gracilis 6 PT':0.15140999999999982,'Euglena longa 2 PT':0.12530300000000016)100:0.31376800000000005,'Lotharella sp. CCMP622':0.449319)100:0.21383300000000016)100:0.270197)84:0.0880209999999999)50:0.018107000000000095,(('Gloeochaete wittrockiana3':0.3962979999999998,'Gloeochaete wittrockiana2':0.08327300000000015)52:0.053160000000000096,('Cyanoptyche gloeocystis1':0.04277799999999976,'Cyanoptyche gloeocystis2':0.02212899999999962)100:0.16056000000000026)58:0.03960000000000008)54:0.05656999999999979,(('Rhodomonas salina3':0.13154399999999988,'Guillardia theta2':0.04964000000000013)95:0.03974999999999973,'Cryptomonas paramecium2':0.14526199999999978)100:0.427508)97:0.138725,(((((((((((sbpChondrus:0.31892299999999985,sbpGaldieria2:0.19284999999999997)97:0.11605200000000027,sbpCyanidioschyzon:0.5246210000000002)85:0.07597299999999985,(sbpEmiliania1:1.1581300000000003,sbpGaldieria:0.47376600000000035)82:0.07718299999999978)96:0.14341000000000026,(((sbpChlamydomonas:0.014845999999999915,sbpVolvox:0.017534999999999634)100:0.09454300000000027,sbpCoccomyxa:0.1350659999999997)97:0.065137,sbpArabidopsis:0.21798099999999998)100:0.28804700000000016)78:0.167767,sbpNannochloropsis:0.9085800000000002)98:0.17170599999999991,(((sbpEmiliania2:0.4229890000000003,sbpEmiliania3:0.15147900000000014)97:0.20577500000000004,sbpOstreococcus:0.1581520000000003)100:0.311763,sbpToxoplasma:0.6328290000000001)100:0.3234079999999997)95:0.10732700000000017,(sbpTrypanosoma:0.7562679999999999,sbpCyanidioschyzon2:0.6635750000000002)84:0.08363299999999985)100:0.7751490000000003,'Diplonema papillatum':1.2187039999999998)89:0.27170099999999975,('Trypanosoma brucei':0.233854,'Leishmania major':0.17471000000000014)100:0.275369)58:0.11631299999999989,((('Chondrus crispus':0.0860369999999997,'Galdieria sulphuraria3':0.2141820000000001)100:0.051741999999999955,'Cyanidioschyzon merolae2':0.12914799999999982)91:0.04824000000000028,'Porphyridium aerugineum2':0.24015699999999995)100:0.09526799999999991)60:0.03988099999999983,('Gloeochaete wittrockiana':0.37178799999999956,'Dictyostelium discoideum':0.3685510000000001)98:0.16424700000000048)61:0.08534000000000042)56:0.033564999999999845,((((((((((((('Euglena longa 4':0.11075200000000018,'Euglena longa 5':0.000002)100:0.02280300000000013,'Euglena gracilis 4':0.007054999999999811)100:0.0437470000000002,'Eutreptiella gymnastica-like CCMP1594 2':0.09976299999999982)100:0.0592649999999999,'Eutreptiella gymnastica-like CCMP1594 3':0.0868190000000002)63:0.021977000000000135,('Emiliania huxleyi':0.19615699999999991,'Eutreptiella gymnastica-like CCMP1594 5':0.06357299999999988)92:0.08167200000000019)31:0.000002,('Euglena longa 6':0.010639999999999983,'Euglena gracilis 2':0.05032300000000012)100:0.07950200000000018)91:0.11016400000000015,(('Skeletonema marinoi':0.021389000000000102,'Thalassiosira pseudonana3':0.050044999999999895)100:0.11949299999999985,'Phaeodactylum tricornutum3':0.06429499999999999)100:0.2486830000000002)97:0.08065999999999995,('Lotharella sp. CCMP6222':0.46784199999999965,'Aureococcus anophagefferens2':0.2100259999999996)65:0.1219800000000002)84:0.1584080000000001,('Phytophtora ramorum':0.13333200000000023,'Phytophtora ramorum2':0.049129999999999896)100:0.1724899999999998)100:0.1021040000000002,'Monosiga brevicollis':0.42358300000000026)89:0.09610099999999999,(((((((('Oryza sativa':0.08854599999999957,'Arabidopsis thaliana':0.030199000000000087)99:0.027324000000000126,'Physcomitrella patens1':0.09769300000000003)97:0.0069509999999999295,('Selaginella moellendorffii1':0.016596999999999973,'Selaginella moellendorffii4':0.000002)100:0.04638600000000004)100:0.050035000000000274,'Selaginella moellendorffii2':0.05751100000000031)100:0.141972,'Coccomyxa subellipsoidea':0.3711750000000005)74:0.030092999999999925,('Ostreococcus tauri2':0.9602250000000003,'Micromonas pusilla2':0.36929900000000027)62:0.13163400000000003)58:0.02289799999999964,'Chlorella variabilis':0.39515900000000004)39:0.014427000000000412,(('Cryptococcus neoformans':0.0742149999999997,'Laccaria bicolor':0.0967640000000003)100:0.14975899999999998,'Aspergillus fumigatus':0.5421209999999999)65:0.12892800000000015)56:0.03264099999999992)63:0.04488799999999982,'Galdieria sulphuraria4':0.8071260000000002)50:0.022387999999999852,(((('Guillardia theta3':0.08943399999999979,'Rhodomonas salina1':0.2186720000000002)100:0.38520999999999983,'Tetrahymena thermophila':0.3070789999999999)89:0.031181000000000125,'Drosophila melanogaster':0.427613)84:0.06958999999999982,'Acanthamoeba castellanii':0.36736900000000006)75:0.03323300000000007)59:0.03770500000000032):0.07927699999999982,((((((('Euglena gracilis 5':0.017386000000000124,'Euglena longa 1':0.03818700000000019)100:0.10832400000000009,'Eutreptiella gymnastica-like CCMP1594 4':0.10041600000000006)100:0.3407300000000002,'Cupriavidus necator':0.32853699999999986)81:0.03979299999999997,'Magnetospirillum magneticum':0.21201099999999995)86:0.035679000000000016,((('Cupriavidus necator2':0.03828200000000015,'Ralstonia solanacearum':0.07962099999999994)100:0.03723799999999988,'Verminephrobacter eiseniae':0.17688199999999998)98:0.04211099999999979,'Burkholderia cenocepacia':0.1798329999999999)100:0.1815190000000002)100:0.3347640000000003,((((('Synechococcus sp. PCC 7335':0.1960449999999998,'Thermosynechococcus elongatus':0.13190800000000014)66:0.05911799999999978,'Cyanothece sp. PCC 7425':0.09604100000000004)66:0.03500099999999984,'Lyngbya sp. PCC 8106':0.1278060000000001)65:0.058803999999999856,('Anabaena variabilis':0.013650000000000162,'Nodularia spumigena':0.017787999999999915)100:0.03747100000000003)100:0.34835800000000017,('Cyanoptyche gloeocystis3':1.793238,'Synechococcus sp. PCC 73352':0.5530400000000002)76:0.20573500000000022)83:0.18491500000000016)58:0.09702699999999975,((('Neospora caninum':0.006352000000000135,'Toxoplasma gondii':0.000002)99:0.016659999999999897,'Eimeria tenella':0.10695299999999985)100:0.21356399999999987,('Perkinsus marinus':0.44943,'Yersinia pestis':0.24291000000000018)45:0.042714999999999836)100:0.31011500000000014)76:0.07927700000000026);

**Glyceraldehyde phosphate dehydrogenase, dataset listed in Data Set S1, Tab 15:**

((((((((((((((((((('Thalassiosira pseudonana':0.000003,'Thalassiosira pseudonana2':0.000003)100:0.05572200000000005,'Skeletonema marinoi':0.04011588780000008)81:0.028784000000000032,'Thalassiosira pseudonana3':0.05250136830000007)100:0.08428599999999997,(('Durinskia baltica2':0.08112541940000007,'Phaeodactylum tricornutum':0.0671700972999999)80:0.035804999999999976,'Phaeodactylum tricornutum2':0.0065558436999999525)80:0.03768499999999997)100:0.06405400000000006,(('Ochromonas sp. CCMP1393':0.0463296809,'Durinskia baltica1':0.07292459759999992)100:0.035978000000000065,'Dinobryon sp. UTEXLB2267':0.038816452100000065)100:0.128927)31:0.024255999999999944,'Aureococcus anophagefferens':0.13696430729999998)29:0.013548000000000004,(('Ectocarpus siliculosus':0.1322594734,'Ectocarpus siliculosus2':0.11794454099999996)78:0.04810499999999995,'Phytophtora ramorum':0.17138284429999995)70:0.04318999999999995)78:0.09727700000000006,(((('Laccaria bicolor':0.04140342149999998,'Laccaria bicolor2':0.06589186759999999)100:0.12331400000000003,'Cryptococcus neoformans':0.09043171679999995)88:0.04127400000000003,('Aspergillus fumigatus':0.11481301919999998,'Aspergillus fumigatus2':0.20973984089999997)100:0.07048600000000005)77:0.03251999999999999,'Drosophila melanogaster':0.195860826)60:0.008301000000000003)49:0.01611600000000002,(((('Arabidopsis thaliana2':0.019468537399999986,'Arabidopsis thaliana3':0.02975680709999995)100:0.060078999999999994,('Oryza sativa':0.011736301699999951,'Oryza sativa6':0.035320910799999994)97:0.018279000000000045)100:0.08600399999999997,'Selaginella moellendorffii':0.10666085719999996)98:0.052100000000000035,('Physcomitrella patens8':0.000002,'Physcomitrella patens9':0.013879531700000003)100:0.10204400000000002)89:0.02954100000000004)61:0.02358899999999997,'Monosiga brevicollis':0.25061763319999997)47:0.009552000000000005,(((((('Perkinsus marinus':0.0041020647999999715,'Perkinsus marinus4':0.032397326000000004)84:0.017974000000000045,'Perkinsus marinus2':0.04490093849999999)100:0.09651900000000002,'Perkinsus marinus3':0.10893468289999997)99:0.031046000000000018,'Neospora caninum':0.2983331683999999)100:0.06768099999999999,(('Chattonella subsalsa':0.1003730745000001,'Heterosigma akashiwo':0.038651048700000024)100:0.13237,'Phytophtora ramorum2':0.17053460369999995)98:0.06147800000000003)99:0.02527299999999999,'Entamoeba histolytica':0.2591058935)76:0.020236000000000032)44:0.017009000000000052,((((((('Pyramimonas amylifera2':0.13729370909999994,'Pyramimonas parkeae2':0.07635991210000004)100:0.07026900000000003,'Chlorella variabilis':0.14560066159999996)75:0.022070000000000034,'Coccomyxa subellipsoidea':0.0862553533)52:0.04236099999999998,(('Chlorella variabilis2':0.18216206359999998,'Coccomyxa subellipsoidea3':0.1623986851)61:0.049634999999999985,'Chlamydomonas reinhardtii2':0.2216520634)56:0.021974999999999967)84:0.04506999999999994,((('Chondrus crispus2':0.14421536840000004,'Cyanidioschyzon merolae2':0.2690183059)72:0.04901500000000003,('Galdieria sulphuraria2':0.12111790700000002,'Rhodella maculata':0.14819737060000004)80:0.029518000000000044)44:0.027436000000000016,'Porphyridium aerugineum2':0.07818751499999999)90:0.04614099999999999)67:0.024685999999999986,((((('Oryza sativa2':0.01746840670000005,'Oryza sativa7':0.08668837330000001)100:0.015225999999999962,'Oryza sativa5':0.004443217299999969)100:0.036082999999999976,'Arabidopsis thaliana5':0.04724303129999996)99:0.05437499999999995,'Selaginella moellendorffii2':0.11173577430000003)99:0.04363600000000001,('Physcomitrella patens':0.033514984199999986,'Physcomitrella patens10':0.01504179579999998)100:0.05415700000000001)99:0.06679000000000002)13:0.018861000000000017,(('Cyanoptyche gloeocystis':0.11676831200000004,'Gloeochaete wittrockiana2':0.09085054790000002)100:0.07971499999999998,'Gloeochaete wittrockiana3':0.15207961969999995)94:0.06635599999999997)9:0.000002)38:0.008677999999999964,((('Dictyostelium discoideum':0.2082410361,'Bathycoccus prasinos3':0.17762288120000003)87:0.04863099999999998,('Guillardia theta':0.03638453399999997,'Rhodomonas salina':0.09277264070000002)100:0.16644199999999998)73:0.03917700000000002,'Micromonas pusilla3':0.22784358380000003)59:0.018940999999999986)65:0.05896800000000002,'Lotharella sp. CCMP622':0.21308649179999994)48:0.018375999999999948,('Porphyromonas gingivalis':0.13972456099999997,'Trypanosoma brucei':0.18338154409999996)97:0.08063299999999995)21:0.008009999999999962,((('Eutreptiella gymnastica-like CCMP1594 1':0.08077856910000003,'Eutreptiella gymnastica NIES-381 3':0.12322109589999997)98:0.05020999999999998,'Euglena longa 2':0.4063773786)100:0.11888200000000004,'Naegleria gruberi':0.21203204669999998)71:0.09956399999999999)35:0.023805999999999994,'Bigelowiella natans':0.23876163839999998)99:0.131231,(('Euglena gracilis 2':0.04038767859999992,'Euglena longa 3':0.02053471809999996)100:0.12470400000000015,('Euglena gracilis 3':0.029687081200000076,'Euglena longa 1':0.012545785899999995)97:0.019628999999999897)100:0.3753900000000001):0.15039899999999995,((((((((((((('Physcomitrella patens4':0.000003,'Physcomitrella patens5':0.000003)100:0.008507000000000042,'Physcomitrella patens3':0.022132972399999895)99:0.00468500000000005,'Physcomitrella patens2':0.005181038099999924)100:0.01252399999999998,('Physcomitrella patens6':0.000003,'Physcomitrella patens7':0.000003)96:0.000003)100:0.03264899999999993,(('Arabidopsis thaliana6':0.037489140299999946,'Oryza sativa4':0.04710140049999989)100:0.019895999999999914,'Arabidopsis thaliana4':0.003416211900000077)100:0.04578400000000005)96:0.022407999999999983,'Selaginella moellendorffii3':0.047152670800000074)100:0.04048199999999991,((('Chlamydomonas reinhardtii':0.042694608399999945,'Volvox carteri f. nagariensis':0.004245143799999962)100:0.030043000000000042,('Dunaliella tertiolecta':0.000002,'Noctiluca scintillans2':0.000003)100:0.09551799999999999)100:0.028362999999999916,'Coccomyxa subellipsoidea2':0.09053610860000005)54:0.027336999999999945)86:0.02639900000000006,(((('Bathycoccus prasinos':0.04468919539999994,'Ostreococcus tauri':0.014721661899999905)85:0.01462399999999997,'Micromonas pusilla2':0.016580099700000073)100:0.08660599999999996,(('Bathycoccus prasinos2':0.06038359000000004,'Ostreococcus tauri2':0.08210435929999993)97:0.030539000000000094,'Micromonas pusilla':0.09036646820000005)100:0.0479989999999999)38:0.022134000000000098,((('Arabidopsis thaliana':0.051433444800000006,'Oryza sativa3':0.039344505499999904)99:0.02736699999999992,'Selaginella moellendorffii4':0.048046216799999986)98:0.021598999999999924,'Pyramimonas amylifera3':0.15114099889999988)100:0.03976399999999991)83:0.011732999999999993)89:0.0367559999999999,((('Cyanoptyche gloeocystis2':0.000003,'Cyanoptyche gloeocystis3':0.004435895300000103)100:0.09308300000000003,'Gloeochaete wittrockiana':0.05053279339999994)90:0.03452199999999994,'Cyanophora paradoxa':0.051239912800000065)100:0.0750050000000001)74:0.015185000000000004,(((((('Porphyridium aerugineum':0.13151494789999996,'Porphyra purpurea':0.14134259139999994)90:0.025023000000000017,'Chondrus crispus':0.10295799690000007)62:0.019729000000000108,'Galdieria sulphuraria':0.1504808032)90:0.02665699999999993,'Rhodella maculata2':0.1257134324)99:0.06085099999999999,'Cyanidioschyzon merolae':0.19214032669999992)78:0.051378000000000035,(('Pyramimonas parkeae':0.029391393999999904,'Pyramimonas parkeae3':0.030284812399999916)73:0.01849400000000001,'Pyramimonas amylifera':0.05205733080000008)100:0.09651599999999994)70:0.018070999999999948)81:0.05328500000000003,((((('Anabaena variabilis':0.03364628230000011,'Nodularia spumigena':0.03409136359999998)100:0.10837999999999992,('Cyanothece sp. PCC 7425':0.03257068919999995,'Thermosynechococcus elongatus':0.0997534391999999)100:0.09965399999999991)85:0.04984199999999994,'Synechococcus sp. PCC 7335':0.20837697519999998)68:0.0158910000000001,(('Paulinella chromatophora':0.09827099419999996,'Prochlorococcus marinus':0.15309134590000006)100:0.18735999999999997,'Crocosphaera watsonii':0.07130313479999995)78:0.030980000000000008)91:0.055169000000000024,'Lyngbya sp. PCC 8106':0.0870566948)100:0.112545)99:0.06402600000000003,(((((('Eutreptiella gymnastica NIES-381 1':0.030677989399999994,'Eutreptiella gymnastica NIES-381 2':0.040970434799999955)100:0.012388999999999983,'Eutreptiella gymnastica NIES-381 4':0.02601348119999991)54:0.011646999999999963,'Eutreptiella gymnastica NIES-381 5':0.02561392320000011)100:0.07001100000000005,('Euglena gracilis 1PT':0.06237797239999998,'Eutreptiella gymnastica-like CCMP1594 2':0.06790787719999991)100:0.04058800000000007)100:0.06228,('Crypthecodinium cohniiP':0.13475968640000002,'Noctiluca scintillans1':0.11278989489999991)100:0.09948199999999996)100:0.06492500000000001,'Oxyrrhis marina':0.5338145014)100:0.07393399999999994)100:0.16216799999999998,'Bacillus anthracis':0.2872151165)100:0.15039900000000006);

**Phosphoglycerate kinase, dataset listed in Data Set S1, Tab 16:**

(((((((((((((((((((('Durinskia baltica':0.030665879600000112,'Kryptoperidinium foliaceum4':0.021590375099999948)100:0.03726800000000008,'Phaeodactylum tricornutum':0.04129386440000005)100:0.028804000000000052,'Thalassiosira pseudonana':0.026647592800000064)99:0.033822999999999936,'Skeletonema marinoi':0.04484207270000007)95:0.04665600000000003,'Noctiluca scintillans2':0.05567403570000007)100:0.15990900000000008,'Aureococcus anophagefferens':0.18968105460000007)98:0.032010000000000094,(('Durinskia baltica3':0.09322199819999999,'Lingulodinium polyedra2':0.055324120599999915)99:0.016192999999999902,'Kryptoperidinium foliaceum3':0.0883137987)100:0.22623700000000002)86:0.04282500000000011,'Ectocarpus siliculosus2':0.0861992775)80:0.014475999999999933,'Vaucheria litorea':0.12835901979999997)89:0.023773999999999962,(('Cryptomonas paramecium2':0.1459782181,'Guillardia theta2':0.04819933550000011)100:0.008912999999999949,('Guillardia theta1':0.05201570420000001,'Rhodomonas salina2':0.06022198259999989)100:0.03216400000000008)100:0.08990000000000009)89:0.03187300000000004,('Chattonella subsalsa':0.027456385000000028,'Heterosigma akashiwo':0.03383132900000008)100:0.050875000000000004)85:0.023676999999999948,((((((('Dinobryon sp. UTEXLB22671':0.041996847200000076,'Ochromonas sp. CCMP1393':0.09077862010000004)99:0.05384699999999998,('Dinobryon sp. UTEXLB22672':0.000003,'Dinobryon sp. UTEXLB22673':0.007244248099999906)100:0.07401900000000006)100:0.10178200000000004,'Ectocarpus siliculosus':0.11503067450000004)69:0.017217000000000038,(('Chondrus crispus':0.059588754600000016,'Porphyra purpurea':0.10509847259999994)99:0.021625999999999923,('Porphyridium aerugineum':0.10060551679999996,'Rhodella maculata':0.08292647129999997)94:0.014796000000000031)99:0.064249)33:0.015867000000000075,('Bigelowiella natans':0.041197753699999895,'Lotharella sp. CCMP622':0.05528038269999991)100:0.092171)75:0.013071999999999973,(('Karenia brevisS2':0.028607059200000062,'Karenia brevisS4':0.047084012200000025)100:0.06429599999999991,('Pleurochrysis carterae2':0.008217336299999989,'Prymnesium parvum Texoma12':0.06730977290000006)100:0.04151199999999999)100:0.043147000000000046)76:0.01374300000000006,('Nannochloropsis gaditana1':0.20772789339999997,'Nannochloropsis gaditana2':0.06545243079999996)99:0.09411999999999998)67:0.01182799999999995)57:0.000002,('Cyanidioschyzon merolae':0.15488740209999996,'Galdieria sulphuraria':0.2254667724999999)99:0.10616599999999998)76:0.04563000000000006,(((((((('Anabaena variabilis':0.016300713399999944,'Nodularia spumigena':0.07253446199999991)100:0.04083700000000001,'Crocosphaera watsonii':0.11661893620000008)93:0.02352299999999996,'Cyanothece sp. PCC 7425':0.08535974940000002)48:0.018291999999999975,('Lyngbya sp. PCC 8106':0.08053120260000002,'Synechococcus sp. PCC 7335':0.12729535600000008)83:0.05152599999999996)46:0.02017000000000002,'Thermosynechococcus elongatus':0.12562924570000011)72:0.03026200000000001,('Paulinella chromatophora':0.06037673890000006,'Prochlorococcus marinus':0.17295040249999993)100:0.08594900000000005)100:0.10901000000000005,((((('Oryza sativa':0.03194477870000001,'Oryza sativa2':0.0220881341000001)100:0.05364600000000008,'Arabidopsis thaliana3':0.06994662760000003)100:0.056486000000000036,'Selaginella moellendorffii2':0.16853129729999994)90:0.011061999999999905,('Arabidopsis thaliana':0.019560816600000086,'Arabidopsis thaliana2':0.01202463400000009)100:0.042596999999999996)96:0.020351999999999926,'Selaginella moellendorffii':0.03642811789999989)100:0.04038300000000006)88:0.041881999999999975,(((('Polytomella parva':0.17647080569999996,'Volvox carteri f. nagariensis':0.0337611914)75:0.007913000000000059,'Chlamydomonas reinhardtii':0.004884838099999955)100:0.03926000000000007,'Dunaliella tertiolecta':0.2180328866000001)96:0.03781600000000007,((('Pyramimonas amylifera':0.0707957292000001,'Pyramimonas parkeae':0.0509621467000001)100:0.07950999999999997,'Chlorella variabilis':0.08060426730000003)87:0.01334499999999994,'Coccomyxa subellipsoidea':0.12443157149999995)86:0.019662999999999986)94:0.024494999999999933)87:0.017152000000000056)78:0.06613100000000005,('Cyanophora paradoxa':0.09488314300000011,'Gloeochaete wittrockiana':0.1289492564999999)91:0.019477000000000078)66:0.019139000000000017,'Cyanoptyche gloeocystis':0.13255010760000008)98:0.058011000000000035,(('Physcomitrella patens':0.05553479760000002,'Physcomitrella patens2':0.07319342130000006)100:0.037717,'Physcomitrella patens3':0.09449717430000004)100:0.12053400000000003)100:0.1848280000000001,(('Bacteroides fragilis':0.057276487700000045,'Porphyromonas gingivalis':0.15187745130000008)100:0.269246,'Flavobacterium columnare':0.36214994080000007)100:0.2386290000000001)73:0.030310999999999755,('Bacillus anthracis':0.3706539753000001,'Listeria monocytogenes':0.5229608721)55:0.051409999999999845)65:0.03026799999999996,(((((((((((('Eutreptiella gymnastica-like CCMP1594 1':0.000003,'Eutreptiella gymnastica-like CCMP1594 4':0.000003)100:0.03838800000000009,'Eutreptiella gymnastica NIES-381 1':0.0687214585)100:0.03155299999999994,('Euglena gracilis 4':0.00768352129999994,'Euglena gracilis 3PT':0.006776149600000059)100:0.0431919999999999)100:0.110908,(((('Guillardia theta3':0.06219247449999998,'Rhodomonas salina':0.05252402190000005)93:0.035813000000000095,'Cryptomonas paramecium':0.07293295090000007)95:0.04507799999999995,'Emiliania huxleyi':0.3682332480000001)74:0.02856600000000009,'Oxyrrhis marina':0.13119874889999994)65:0.017622000000000027)74:0.016378000000000004,('Emiliania huxleyi2':0.015147489799999914,'Emiliania huxleyi3':0.000003)100:0.286138)75:0.02747799999999989,(('Pleurochrysis carterae3':0.1021166402,'Prymnesium parvum Texoma1':0.13728192950000007)90:0.035420999999999925,'Pleurochrysis carterae':0.11463107100000003)65:0.02822199999999997)48:0.022407000000000066,(('Symbiodinium sp. CCMP4211':0.04950776689999992,'Symbiodinium sp. CCMP4214':0.05629302040000006)100:0.07966899999999999,'Durinskia baltica2':0.16912363419999998)94:0.046221999999999985)54:0.035744999999999916,(((((('Karenia brevisS3':0.09562415289999993,'Karenia brevisS6':0.16331370320000005)68:0.0250999999999999,'Karenia brevisS5':0.09719401249999993)71:0.039309999999999956,'Kryptoperidinium foliaceum1':0.054291290899999955)63:0.01527400000000001,('Lingulodinium polyedra1':0.11774873709999989,'Lingulodinium polyedra3':0.02861367700000006)88:0.021309000000000022)73:0.020590000000000108,('Crypthecodinium cohnii':0.10560663850000007,'Noctiluca scintillans1':0.06889416589999997)100:0.14368099999999995)62:0.02168900000000007,('Symbiodinium sp. CCMP4212':0.043648585599999956,'Symbiodinium sp. CCMP4215':0.05037811709999995)83:0.015594000000000108)84:0.05071899999999996)30:0.03252699999999997,(((('Symbiodinium sp. CCMP4213':0.047957427899999905,'Symbiodinium sp. CCMP4216':0.016188470999999982)100:0.039323,'Crypthecodinium cohnii2':0.10194836809999996)99:0.029274000000000022,('Karenia brevisS1':0.04025845639999992,'Karenia brevisS7':0.017914468699999908)100:0.09646700000000008)64:0.010434999999999972,('Kryptoperidinium foliaceum2':0.1163206416,'Kryptoperidinium foliaceum5':0.06715660579999994)97:0.021239000000000008)99:0.03154799999999991)100:0.16598800000000002,('Eutreptiella gymnastica-like CCMP1594 3':0.14380870690000003,'Phytophtora ramorum':0.17555410449999997)100:0.04446400000000006)98:0.09722900000000001,(('Euglena gracilis 2':0.019368422499999927,'Euglena longa 1':0.0936283752)100:0.334284,('Naegleria gruberi':0.46789507539999997,'Laccaria bicolor':0.2058643548000001)79:0.08739699999999995)70:0.022035000000000027)100:0.2986329999999999,((((('Euglena gracilis 1B':0.03190014969999999,'Euglena gracilis 1':0.000003)100:0.02784399999999998,'Euglena longa 2':0.01660041379999999)100:0.052000999999999964,('Eutreptiella gymnastica-like CCMP1594 2':0.11269512339999999,'Eutreptiella gymnastica NIES-381 2':0.08791770850000002)100:0.039795000000000025)100:0.2972159999999999,((('Trypanosoma brucei':0.00746156259999986,'Trypanosoma brucei3':0.16204816099999986)92:0.009740000000000082,'Trypanosoma brucei2':0.012119018599999976)99:0.13055700000000003,'Leishmania major1':0.09884054080000015)100:0.1950860000000001)100:0.1128610000000001,'Leishmania major2':0.8454950784999999)99:0.14687300000000003):0.030268000000000184);

**Phosphoribulokinase, dataset listed in Data Set S1, Tab 17:**

((((((((((('Guillardia theta':0.15596538319999997,'Rhodomonas salina':0.2352551476)98:0.08298499999999998,'Cryptomonas paramecium':0.27971967650000007)100:0.16249599999999997,('Phaeodactylum tricornutum':0.15643176859999997,'Thalassiosira pseudonana':0.15039566930000003)84:0.12344500000000003)92:0.06319699999999995,'Emiliania huxleyi':0.5525154438)50:0.07617700000000005,(((('Euglena gracilis':0.02425356550000002,'Euglena longa':0.05964046940000001)97:0.060953000000000035,'Eutreptiella gymnastica-like CCMP1594':0.06498231160000001)47:0.05077200000000004,'Eutreptiella gymnastica NIES-381':0.05819824149999997)100:0.42819299999999993,'Ectocarpus siliculosus':0.10996973489999995)81:0.06657100000000005)89:0.07881900000000008,(('Bathycoccus prasinos':0.06808705000000004,'Ostreococcus tauri':0.046814661300000004)71:0.011155999999999944,'Micromonas pusilla':0.027853968399999984)100:0.08160900000000004)44:0.03805499999999995,(((('Chlamydomonas reinhardtii':0.02106741550000013,'Volvox carteri f. nagariensis':0.020189970400000035)100:0.02940900000000002,'Dunaliella tertiolecta':0.11402544650000013)93:0.01048899999999997,'Chlorella variabilis':0.07429665480000003)100:0.051775000000000015,((('Pyramimonas parkeae':0.000003,'Pyramimonas parkeae2':0.1606920464)100:0.053921999999999914,'Pyramimonas amylifera':0.03300271619999995)99:0.05607899999999999,'Coccomyxa subellipsoidea':0.08343717299999998)91:0.04861900000000008)30:0.014168999999999987)47:0.026939000000000046,((('Oryza sativa':0.02585452340000005,'Oryza sativa2':0.11100106030000001)86:0.008682000000000079,'Arabidopsis thaliana':0.0419007374)100:0.03394799999999987,(('Selaginella moellendorffii':0.000003,'Selaginella moellendorffii2':0.003870475100000048)100:0.03197399999999995,'Physcomitrella patens':0.04238081260000004)70:0.00789799999999996)97:0.023694000000000104)100:0.136563,(((('Cyanoptyche gloeocystis2':0.000003,'Cyanoptyche gloeocystis3':0.008080745300000025)100:0.055709999999999926,'Cyanoptyche gloeocystis':0.0350837651)99:0.05484,'Gloeochaete wittrockiana':0.08069170530000003)85:0.026403000000000065,'Gloeochaete wittrockiana2':0.38582231990000004)99:0.06507600000000002)76:0.027302999999999966,(((('Chondrus crispus':0.24397860919999992,'Rhodella maculata':0.10491793439999997)99:0.08284400000000003,'Galdieria sulphuraria':0.13596538999999996)92:0.04979,'Lotharella sp. CCMP622':0.6467240496)80:0.018982000000000054,'Cyanidioschyzon merolae':0.3351941785999999)92:0.037864999999999926):0.04141300000000003,(((('Anabaena variabilis':0.03880748860000005,'Nodularia spumigena':0.04779570430000002)100:0.13451600000000008,('Cyanothece sp. PCC 7425':0.051592984699999955,'Thermosynechococcus elongatus':0.12170888280000003)94:0.05369600000000008)39:0.014793999999999974,('Crocosphaera watsonii':0.12095143419999999,'Synechococcus sp. PCC 7335':0.1006569504)71:0.02967300000000006)56:0.033911000000000024,'Lyngbya sp. PCC 8106':0.10802618970000011)100:0.04141299999999992);

**Ribulose phosphate epimerase, dataset listed in Data Set S1, Tab 18:**

((((((((((((((((((('Euglena gracilis2':0.029803000000000024,'Euglena longa1':0.09234600000000004)100:0.06878899999999999,'Nannochloropsis gaditana':0.21536599999999995)43:0.04009600000000013,('Eutreptiella gymnastica-like CCMP1594 2':0.14140200000000003,'Eutreptiella gymnastica NIES-381 2':0.07642799999999994)37:0.018836000000000075)68:0.0501459999999998,'Phytophtora ramorum':0.18699699999999986)88:0.10418600000000011,(('Cryptomonas paramecium1':0.017179999999999973,'Guillardia theta':0.26991300000000007)99:0.06569099999999994,'Rhodomonas salina1':0.21357800000000005)100:0.14700599999999997)40:0.03428000000000009,(((('Euglena gracilis1 PT':0.01215300000000008,'Eutreptiella gymnastica-like CCMP1594 1':0.10397600000000007)54:0.007885999999999838,'Euglena longa2 PT':0.05145399999999989)55:0.0455509999999999,'Eutreptiella gymnastica NIES-381 1':0.11541499999999982)100:0.14921300000000004,'Aureococcus anophagefferens1':0.455279)56:0.0193500000000002)38:0.08402200000000004,'Phaeodactylum tricornutum1':0.374231)33:0.014346999999999888,'Emiliania huxleyi2':0.3928099999999999)55:0.03425300000000009,(('Bathycoccus prasinos2':0.13975099999999996,'Micromonas pusilla2':0.35589099999999996)98:0.060462000000000016,'Ostreococcus tauri':0.18534200000000012)97:0.12658800000000014)51:0.040349999999999886,'Perkinsus marinus':0.550254)42:0.03325400000000012,('Pyramimonas amylifera1':0.2465599999999999,'Pyramimonas parkeae1':0.14924300000000001)97:0.16375400000000018)34:0.027774999999999883,'Aspergillus fumigatus':0.4531910000000001)33:0.037336000000000036,(((((('Homo sapiens':0.06359200000000009,'Xenopus laevis':0.026923999999999948)96:0.06216899999999992,'Danio rerio':0.07747900000000008)98:0.13780400000000004,('Crassostrea gigas':0.15728200000000014,'Schistosoma haematobium':0.46969700000000003)94:0.086144)67:0.03415799999999991,('Acyrthosiphon pisum':0.33798799999999996,'Drosophila melanogaster':0.14215599999999995)100:0.10403300000000004)96:0.19003900000000007,('Paramecium tetraurelia':0.5362259999999999,'Tetrahymena thermophila':0.19930000000000003)99:0.164895)62:0.06631399999999998,(('Neospora caninum':0.0444739999999999,'Toxoplasma gondii':0.08140799999999992)100:0.27813200000000005,'Picrophilus torridus':1.302514)57:0.19637799999999994)55:0.12780900000000006)48:0.08500300000000016,((((((('Entamoeba histolytica':0.5207830000000002,'Chlorella variabilis3':0.2540610000000001)59:0.10502699999999976,'Gloeochaete wittrockiana':0.2832279999999998)55:0.10712500000000014,'Acanthamoeba castellanii':0.20172000000000012)27:0.05143000000000009,((('Arabidopsis thaliana2':0.11131999999999986,'Oryza sativa2':0.08417000000000008)100:0.04288599999999998,'Selaginella moellendorffii1':0.1353740000000001)100:0.08320000000000016,'Physcomitrella patens2':0.1005060000000002)100:0.12442500000000001)24:0.022028999999999854,(((('Bigelowiella natans3':0.21081700000000003,'Lotharella sp. CCMP622':0.11680400000000013)100:0.07850899999999994,('Cryptococcus neoformans':0.42039800000000005,'Laccaria bicolor':0.18516700000000008)100:0.5229049999999997)51:0.0594260000000002,('Dictyostelium discoideum':0.27708299999999997,'Naegleria gruberi':0.39076100000000014)59:0.12712800000000013)45:0.07181099999999985,'Ectocarpus siliculosus2':0.438075)24:0.04362200000000005)47:0.04151499999999975,(('Chlamydomonas reinhardtii2':0.07479000000000013,'Volvox carteri f. nagariensis':0.14552699999999996)99:0.07933999999999974,'Dunaliella tertiolecta2':0.2659609999999999)96:0.08815199999999979)45:0.03463200000000022,'Porphyridium aerugineum1':0.3483379999999998)28:0.0284810000000002)35:0.0406979999999999,(((('Leptomonas pyrrhocoris':0.13918699999999995,'Leishmania major':0.10441699999999998)100:0.457951,'Trypanosoma brucei':0.2253989999999999)98:0.08726999999999996,'Cyanoptyche gloeocystis1':0.5676029999999999)55:0.07354499999999975,'Leishmania major2':0.8319279999999998)34:0.07672300000000032)39:0.03758000000000017,(((('Ostreococcus tauri2':0.1917629999999999,'Pyramimonas amylifera2':0.46593399999999985)73:0.10119100000000003,'Micromonas pusilla1':0.21426999999999996)100:0.19645700000000033,('Chlorella variabilis1':0.5043040000000001,'Ectocarpus siliculosus3':0.7885550000000001)92:0.10746800000000012)74:0.09797600000000006,'Cyanidioschyzon merolae':0.5555900000000003)43:0.031166999999999945)44:0.06325299999999956,'Chondrus crispus':0.3191639999999998)39:0.01948300000000014,('Corynebacter diphteriae':0.3978189999999999,'Mycobacterium tuberculosis':0.2782859999999998)100:0.24725100000000033):0.20695450000000015,((((((((((((('Bathycoccus prasinos':0.056942999999999966,'Micromonas pusilla3':0.04862999999999995)100:0.06381900000000007,'Dunaliella tertiolecta':0.11565599999999998)97:0.05014699999999994,'Pyramimonas amylifera3':0.057477)46:0.0164979999999999,('Chlorella variabilis2':0.0744689999999999,'Coccomyxa subellipsoidea':0.13423999999999991)93:0.05209099999999989)46:0.02538499999999999,('Polytomella parva':0.2497720000000001,'Pyramimonas parkeae2':0.03047399999999989)56:0.01995900000000006)65:0.030977000000000032,(((('Physcomitrella patens1':0.015273000000000092,'Physcomitrella patens3':0.014985000000000026)100:0.03815600000000008,'Arabidopsis thaliana':0.000002)100:0.021001000000000047,'Oryza sativa1':0.03148100000000009)99:0.034129000000000076,'Selaginella moellendorffii2':0.04916900000000002)99:0.04627200000000009)66:0.04443799999999998,'Chlamydomonas reinhardtii':0.1508449999999999)78:0.016858999999999957,(('Galdieria sulphuraria':0.11709199999999997,'Rhodella maculata':0.133343)88:0.07949800000000007,'Porphyridium aerugineum2':0.11243399999999992)86:0.06270599999999993)84:0.03465600000000002,'Gloeochaete wittrockiana2':0.10393399999999997)80:0.02787099999999998,((((((('Cyanothece sp. PCC 7425':0.0408980000000001,'Thermosynechococcus elongatus':0.02040799999999998)100:0.04574199999999995,'Crocosphaera watsonii':0.03262900000000002)81:0.029905999999999988,'Lyngbya sp. PCC 8106':0.012923000000000018)78:0.025060000000000082,'Synechococcus sp. PCC 7335':0.08963999999999994)93:0.027039000000000035,'Nodularia spumigena':0.06000300000000003)66:0.005044999999999966,'Anabaena variabilis':0.000002)100:0.20199499999999992,('Cyanoptyche gloeocystis2':0.000002,'Cyanoptyche gloeocystis3':0.015260999999999969)100:0.10131800000000002)90:0.06912399999999996)98:0.12207100000000004,'Cyanidioschyzon merolae2':0.16532499999999994)98:0.10902000000000012,(((((((((((((('Phaeodactylum tricornutum2':0.0503579999999999,'Thalassiosira pseudonana1':0.07356800000000008)98:0.039365000000000094,'Thalassiosira pseudonana2':0.008529999999999927)100:0.061434999999999906,'Rhodomonas salina2':0.06275300000000006)30:0.015384999999999982,'Guillardia theta2':0.07459299999999991)67:0.013965999999999923,'Cryptomonas paramecium2':0.1543460000000001)97:0.025644999999999918,(('Bigelowiella natans2':0.036243000000000025,'Lotharella sp. CCMP622 2':0.04331900000000011)70:0.015042999999999918,'Bigelowiella natans1':0.7873540000000001)88:0.07315099999999997)88:0.07494099999999992,'Aureococcus anophagefferens2':0.000002)76:0.023276000000000074,'Emiliania huxleyi':0.10868199999999995)90:0.047552999999999956,'Ectocarpus siliculosus1':0.11059400000000008)90:0.10314500000000004,'Shewanella baltica':0.07072999999999996)89:0.08025299999999991,'Burkholderia cenocepacia':0.04574999999999996)87:0.0635730000000001,'Ralstonia solanacearum':0.09305500000000011)85:0.06096699999999999,'Amphimedon queenslandica':0.30154599999999987)100:0.3367530000000001,(('Magnetospirillum magneticum':0.19289599999999996,'Nitrobacter hamburgensis':0.2931079999999999)97:0.06466400000000005,'Rhodobacter sphaeroides':0.34107599999999993)95:0.09326900000000005)62:0.061741000000000046)66:0.09133400000000003,(((('Bacteroides fragilis':0.3593709999999999,'Prevotella ruminicola':0.21726500000000004)100:0.15353299999999992,'Flavobacterium columnare':0.40093)100:0.19212300000000004,('Listeria monocytogenes':0.4910650000000001,'Staphyllococcus aureus':0.608244)64:0.09761200000000003)97:0.07321200000000005,'Listeria monocytogenes2':0.231309)95:0.09764799999999996)62:0.2069544999999997);

**Ribose-phosphate isomerase, dataset listed in Data Set S1, Tab 19:**

(('Picrophilus torridus':0.5170400000000002,'Thermoplasma volcanium':0.45028000000000024):0.4219499999999998,(((('Acanthamoeba castellanii':0.35013000000000005,('Monosiga brevicollis':0.2967599999999999,'Gloeochaete wittrockiana3':0.2896300000000003)49:0.21989999999999998)44:0.07894999999999985,'Drosophila melanogaster':0.39154)78:0.02452999999999994,('Dictyostelium discoideum':0.4430200000000002,(('Lotharella sp. CCMP6222':0.21668999999999983,('Phytophtora ramorum':0.07853000000000021,'Pythium ultimum var. sporangiiferum':0.09296000000000015)100:0.2545099999999998)89:0.04164000000000012,('Aspergillus fumigatus':0.8693499999999998,('Cryptococcus neoformans':0.11847999999999992,'Laccaria bicolor':0.2179500000000001)100:0.35928000000000004)95:0.1942700000000004)88:0.10938999999999988)88:0.04674999999999985)100:0.3964700000000003,('Staphyllococcus aureus':1.3116699999999994,(((((('Eimeria tenella':0.7766099999999998,('Euglena gracilis 1':0.0685699999999998,'Euglena longa 3':0.08840000000000003)100:0.8842399999999997)89:0.13149999999999995,(('Cyanoptyche gloeocystis2':0.16519999999999957,'Cyanoptyche gloeocystis3':0.3183499999999997)100:0.3634900000000001,('Gloeochaete wittrockiana4':0.7320099999999998,'Gloeochaete wittrockiana':0.5073599999999998)88:0.10485999999999995)99:0.3252299999999999)61:0.01802000000000037,((('Neospora caninum':0.0014899999999999913,'Toxoplasma gondii':0.08679999999999977)100:0.39561,(((('Lotharella sp. CCMP622':0.22639999999999993,(((((('Bathycoccus prasinos':0.029380000000000184,('Ostreococcus tauri':0.05292999999999992,(((('Cryptomonas paramecium1':0.03986000000000001,('Guillardia theta':0.14525999999999994,'Rhodomonas salina':0.10926999999999998)100:0.13511000000000006)53:0.034030000000000005,'Guillardia theta2':0.04374000000000011)44:0.022930000000000117,'Cryptomonas paramecium2':0.18043999999999993)99:0.0944199999999995,((('Euglena gracilis 2 PT':0.012359999999999705,('Euglena longa 1':0.02491000000000021,'Euglena longa 2 PT':0.0)100:0.04211999999999971)76:0.01330000000000009,'Eutreptiella gymnastica NIES-381':0.10057999999999989)49:0.02245000000000008,('Eutreptiella gymnastica-like CCMP1594 1':0.0,'Eutreptiella gymnastica-like CCMP1594 2':0.0)100:0.019979999999999887)100:0.12277000000000005)87:0.01097999999999999)72:0.04611000000000054)65:0.009339999999999904,'Micromonas pusilla':0.0387000000000004)81:0.053690000000000015,('Pyramimonas amylifera1':0.09359000000000028,'Pyramimonas parkeae':0.027369999999999894)97:0.03376000000000001)78:0.03753999999999991,'Thalassiosira pseudonana2':0.011569999999999858)78:0.029290000000000038,'Thalassiosira pseudonana':0.15418999999999983)47:0.007330000000000059,'Phaeodactylum tricornutum':0.17528000000000032)62:0.017129999999999868)54:0.031179999999999986,('Emiliania huxleyi':0.20197999999999983,'Aureococcus anophagefferens':0.13804999999999978)59:0.04097000000000017)100:0.07816000000000001,((((('Chondrus crispus2':0.12809000000000026,('Galdieria sulphuraria2':0.20448000000000022,'Galdieria sulphuraria':0.08115000000000006)100:0.18116999999999983)65:0.054320000000000146,'Porphyridium aerugineum':0.06529000000000007)59:0.01134999999999975,'Rhodella maculata':0.08765999999999963)73:0.0933900000000003,'Nannochloropsis gaditana':0.12115000000000009)29:0.0,'Ectocarpus siliculosus':0.07479000000000013)73:0.05100999999999978)78:0.035719999999999974,'Cyanidioschyzon merolae':0.12212999999999985)88:0.10804999999999998)49:0.04300999999999977,(((((((('Chlamydomonas reinhardtii':0.013650000000000162,'Dunaliella tertiolecta':0.1500600000000003)77:0.011800000000000033,'Volvox carteri f. nagariensis':0.018090000000000384)78:0.02450999999999981,'Chlorella variabilis2':0.04557000000000011)63:0.02253000000000016,'Polytomella parva':0.10616000000000003)89:0.041309999999999736,('Coccomyxa subellipsoidea2':0.01831999999999967,'Coccomyxa subellipsoidea1':0.01546000000000003)100:0.08589000000000002)97:0.10637000000000008,(('Cyanoptyche gloeocystis':0.18721999999999994,'Gloeochaete wittrockiana2':0.12851999999999997)79:0.10705000000000009,((('Arabidopsis thaliana3':0.23591999999999969,'Oryza sativa3':0.07320999999999955)70:0.07705000000000028,'Selaginella moellendorffii1':0.0869500000000003)64:0.031629999999999825,('Physcomitrella patens2':0.0,'Physcomitrella patens3':0.01664000000000021)100:0.05013999999999985)64:0.06213000000000024)69:0.0384199999999999)71:0.054159999999999986,(('Arabidopsis thaliana2':0.06810000000000027,'Oryza sativa2':0.3959800000000002)97:0.07235999999999976,'Arabidopsis thaliana1':0.13293)97:0.22890999999999995)97:0.12685999999999975,'Aureococcus anophagefferens2':1.5121599999999997)17:0.0)82:0.12988000000000044)64:0.06388999999999978,((('Chlamydomonas reinhardtii2':1.5492799999999995,('Oryza sativa1':0.9206799999999999,'Physcomitrella patens1':0.3695999999999997)100:0.36064000000000007)88:0.1807000000000003,'Chlorella variabilis':1.48826)78:0.12475000000000014,'Pyramimonas amylifera2':1.2974000000000006)95:0.5643499999999997)66:0.08226000000000022,('Listeria monocytogenes':0.7982200000000002,('Nitrobacter hamburgensis':0.3365,'Rhodobacter sphaeroides':0.5265900000000001)100:0.20714000000000032)80:0.11556999999999995)56:0.11067999999999989,((('Paulinella chromatophora':0.18005000000000004,'Prochlorococcus marinus':0.16705999999999976)100:0.28309000000000006,((((((('Anabaena variabilis':0.03642999999999974,'Nodularia spumigena':0.02475000000000005)100:0.09047000000000027,'Crocosphaera watsonii':0.1705700000000001)63:0.02635999999999994,'Lyngbya sp. PCC 8106':0.09296999999999978)59:0.03716000000000008,'Synechococcus sp. PCC 7335':0.1588099999999999)96:0.10494000000000003,'Cyanothece sp. PCC 7425':0.12725000000000009)100:0.09871000000000008,'Thermosynechococcus elongatus':0.08423999999999987)100:0.1787399999999999,(('Physcomitrella patens4':0.10786000000000007,'Physcomitrella patens5':0.09074999999999989)100:0.02076000000000011,'Selaginella moellendorffii2':0.13811000000000018)100:0.40932999999999975)99:0.09065000000000012)99:0.18823999999999996,('Pyrobaculum aerophilum':0.96055,('Sulfolobus tocodaii':1.7153299999999998,((((('Burkholderia cenocepacia':0.16561000000000026,('Cupriavidus necator':0.0690900000000001,'Ralstonia solanacearum':0.10263)100:0.20176000000000016)94:0.23673999999999973,'Verminephrobacter eiseniae':0.29434000000000005)87:0.25644,'Vibrio cholerae':0.11768)59:0.05457000000000001,'Shewanella baltica':0.09065999999999974)98:0.5327999999999999,'Chondrus crispus':3.17174)33:0.03011000000000008)63:0.18101000000000012)61:0.20764000000000005)70:0.07179000000000002)57:0.09296999999999978)53:0.04167000000000032):0.02220999999999984);

**Sedoheptulose bisphosphatase, dataset listed in Data Set S1, Tab 20:**

(((((((((((('Chlamydomonas reinhardtii':0.025648705400000082,'Volvox carteri f. nagariensis':0.013229439699999901)98:0.06462900000000005,('Chlorella variabilis2':0.1437296157000001,'Coccomyxa subellipsoidea':0.15359739109999992)64:0.026918999999999915)56:0.037806000000000006,'Dunaliella tertiolecta2':0.21121197539999992)63:0.0708390000000001,(('Euglena gracilis':0.009140148399999992,'Euglena longa':0.0564159273)100:0.19108999999999998,'Eutreptiella gymnastica NIES-381 2':0.1625442641000001)100:0.19846199999999992)57:0.03837399999999991,((('Physcomitrella patens':0.015267213500000043,'Physcomitrella patens2':0.023382119599999918)100:0.042988000000000026,'Selaginella moellendorffii':0.09452794180000001)99:0.035215999999999914,('Arabidopsis thaliana':0.04610357510000007,'Oryza sativa':0.0712128487999999)99:0.04273400000000005)100:0.1649210000000001)100:0.2118389999999999,(('Cyanoptyche gloeocystis':0.005320240399999898,'Cyanoptyche gloeocystis2':0.000002)100:0.17741099999999999,'Gloeochaete wittrockiana':0.1192653023000001)100:0.12821100000000007)93:0.14106700000000005,'Emiliania huxleyi1':1.1045736393)7:0.02599399999999985,((((('Lotharella sp. CCMP622':0.14591039449999998,'Lotharella sp. CCMP6222':0.5025148578)58:0.051415999999999906,'Ectocarpus siliculosus':0.18401767260000002)56:0.09315700000000016,(('Chondrus crispus':0.1630826824,'Porphyridium aerugineum2':0.14853726849999993)96:0.09540100000000007,'Rhodella maculata':0.13453714890000001)87:0.05380600000000002)24:0.03714700000000004,'Galdieria sulphuraria2':0.2332622577000001)46:0.08005299999999993,('Cyanidioschyzon merolae':0.4010684322999998,'Galdieria sulphuraria':0.46697512529999985)27:0.04978600000000011)11:0.0369489999999999)83:0.1394540000000002,('Cryptomonas paramecium':0.6466435332999998,'Rhodomonas salina':0.33492343319999973)96:0.13051100000000027)86:0.076241,(((((((((('Eutreptiella gymnastica-like CCMP1594 1':0.0052283186999999565,'Eutreptiella gymnastica-like CCMP1594 2':0.010484079299999838)100:0.008337000000000039,'Eutreptiella gymnastica-like CCMP1594 3':0.07310842439999998)100:0.029787000000000008,'Eutreptiella gymnastica NIES-381 1':0.1006061573999999)100:0.081596,'Guillardia theta2':0.07228276860000005)98:0.05427399999999993,'Cryptomonas paramecium2':0.11374647579999975)95:0.04022900000000007,'Guillardia theta':0.2941546532000001)99:0.11744500000000002,((('Bathycoccus prasinos':0.1522249255000001,'Ostreococcus tauri':0.07617741139999978)98:0.04032299999999989,('Micromonas pusilla':0.1212513584999999,'Pyramimonas parkeae':0.09772713519999998)98:0.04905999999999988)93:0.03894399999999987,'Phaeodactylum tricornutum':0.14191840779999954)98:0.07376100000000019)57:0.03922800000000004,('Emiliania huxleyi2':0.36921574469999996,'Emiliania huxleyi3':0.16173551979999967)99:0.14161400000000013)100:0.24824100000000016,('Eimeria tenella':0.47506808870000006,'Toxoplasma gondii':0.36583772979999996)100:0.28331099999999987)100:0.2745869999999999,('Porphyridium aerugineum':0.6276236974000002,'Rhodella maculata2':0.9478545191000001)88:0.07255300000000009)88:0.04157300000000008)82:0.1066959999999999,(((('Dunaliella tertiolecta':0.32562769829999993,'Volvox carteri f. nagariensis2':0.3419175282)100:0.21434700000000007,'Chlorella variabilis':0.45326467959999994)100:0.28153300000000003,(('Ectocarpus siliculosus2':0.2920419042,'Nannochloropsis gaditana':0.5556541062)91:0.04565700000000006,'Thalassiosira pseudonana':0.5009425613)100:0.28509000000000007)73:0.07056399999999963,('Trypanosoma brucei':0.8729297035000001,'Cyanidioschyzon merolae2':0.8205055197000002)81:0.03648999999999969)68:0.036159000000000496):0.5536830000000001,((((((('FBP_Cryptococcus neoformans':0.10029333439999988,'FBP_Laccaria bicolor':0.07212105039999983)100:0.17007400000000006,'FBP_Aspergillus fumigatus':0.4857297974999999)38:0.06260600000000016,'FBP_Emiliania huxleyi':0.7321159686)37:0.06958999999999982,'FBP_Tetrahymena thermophila':0.3503020937999999)42:0.06763800000000009,((('FBP_Chondrus crispus':0.13489096890000019,'FBP_Galdieria sulphuraria':0.15424510970000016)97:0.12614100000000006,'FBP_Dictyostelium discoideum':0.47004868899999996)97:0.06562100000000015,'FBP_Trypanosoma brucei':0.5764892642000001)43:0.08172099999999993)69:0.03588799999999992,'FBP_Arabidopsis thaliana':0.4613305413000002)92:0.11647600000000002,((('FBP_Synechococcus sp. PCC 7335':0.15430940009999983,'FBP_Thermosynechococcus elongatus':0.1802098341999998)89:0.07044499999999987,'FBP_Anabaena variabilis':0.10001159059999987)100:0.3610899999999999,'FBP_Synechococcus sp. PCC 7335_2':0.6178952746999999)98:0.291639)100:0.5536830000000001);

**Transketolase, dataset listed in Data Set S1, Tab 21:**

(((((((((((((((((('Arabidopsis thaliana2':0.043862999999999985,'Arabidopsis thaliana1':0.04446799999999973)100:0.044877000000000056,('Oryza sativa2':0.057926000000000144,'Oryza sativa1':0.1531880000000001)100:0.026147999999999616)100:0.05328500000000025,'Selaginella moellendorffii2':0.09952200000000033)91:0.02861899999999995,('Physcomitrella patens2':0.06205999999999978,'Physcomitrella patens1':0.08019799999999977)99:0.027982000000000173)100:0.06590300000000004,('Physcomitrella patens3':0.20058299999999996,'Selaginella moellendorffii':0.24032900000000001)100:0.40313500000000024)94:0.04045999999999994,('Pyramimonas amylifera':0.0824720000000001,'Pyramimonas parkeae':0.029886999999999997)100:0.1381920000000001)92:0.03292599999999979,((((('Bathycoccus prasinos':0.10437300000000027,'Micromonas pusilla':0.07654499999999986)100:0.03238500000000011,'Ostreococcus tauri':0.33103000000000016)100:0.12325399999999975,'Coccomyxa subellipsoidea':0.1380490000000001)99:0.06339400000000017,'Chlorella variabilis':0.16334099999999996)100:0.06275899999999979,((('Chlamydomonas reinhardtii':0.0668899999999999,'Volvox carteri f. nagariensis':0.05227499999999985)100:0.05518400000000012,'Polytomella parva':0.14554899999999993)100:0.027125999999999983,'Dunaliella tertiolecta':0.1520100000000002)100:0.047787000000000024)99:0.04396299999999975)91:0.04261700000000035,(((('Chondrus crispus2':0.5833690000000002,'Chondrus crispus':0.1823640000000002)79:0.056379999999999875,'Rhodella maculata':0.18625500000000006)77:0.05995999999999979,'Porphyridium aerugineum':0.2575389999999995)62:0.05393300000000023,(('Cyanidioschyzon merolae2':0.02318700000000007,'Cyanidioschyzon merolae':0.0986290000000003)100:0.2771880000000002,'Galdieria sulphuraria':0.2784880000000003)83:0.057386999999999855)99:0.08695800000000009)84:0.03416599999999992,(('Cyanoptyche gloeocystis2':0.0229919999999999,'Cyanoptyche gloeocystis1':0.016970999999999847)100:0.1187560000000003,'Gloeochaete wittrockiana1':0.1933360000000004)100:0.08970999999999973)91:0.03433799999999998,(((((('Anabaena variabilis':0.07005599999999967,'Nodularia spumigena':0.03993000000000002)100:0.03224000000000027,'Crocosphaera watsonii':0.07004700000000019)100:0.06631900000000002,'Thermosynechococcus elongatus':0.09764700000000026)93:0.02894099999999966,(('Paulinella chromatophora':0.08408400000000027,'Prochlorococcus marinus':0.11878000000000011)100:0.1825909999999995,'Synechococcus sp. PCC 7335':0.13644799999999968)89:0.02988400000000002)84:0.013717000000000201,'Cyanothece sp. PCC 7425':0.11509299999999989)91:0.0408059999999999,'Lyngbya sp. PCC 8106':0.14715100000000003)93:0.028690000000000104)90:0.06239600000000012,((('Euglena gracilis 3 PT':0.017115999999999687,'Euglena longa 1 PT':0.0567479999999998)100:0.05257200000000006,('Eutreptiella gymnastica-like CCMP1594 1':0.05071700000000012,'Eutreptiella gymnastica NIES-381 2':0.1019779999999999)100:0.0209769999999998)100:0.05778099999999986,('Euglena gracilis 1':0.08574700000000002,'Euglena longa 3':0.06086400000000003)100:0.14220299999999986)100:0.21214400000000033)94:0.05644199999999966,(((((((('Diplonema papillatum':0.38201399999999985,'Cryptococcus neoformans2':0.7569689999999998)91:0.060203000000000007,('Trypanosoma brucei':0.2580860000000005,'Leishmania major':0.24421500000000052)100:0.20371099999999975)93:0.05155100000000035,('Naegleria gruberi1':0.3438870000000003,'Naegleria gruberi2':0.23761900000000047)100:0.35460899999999995)94:0.05940299999999965,(('Cryptococcus neoformans':0.21118799999999993,'Laccaria bicolor':0.25302099999999994)86:0.06339799999999984,'Aspergillus fumigatus':0.26766500000000004)100:0.19329600000000013)44:0.030829999999999913,(((('Perkinsus marinus3':0.06509399999999976,'Perkinsus marinus1':0.058807000000000276)96:0.050756000000000245,'Perkinsus marinus2':0.07874500000000051)100:0.29847599999999996,('Neospora caninum':0.1494620000000002,'Toxoplasma gondii':0.054088000000000136)100:0.4197009999999999)86:0.0972789999999999,(('Lotharella sp. CCMP6222':0.17334300000000002,'Lotharella sp. CCMP622':0.23589099999999963)100:0.19462800000000025,'Gloeochaete wittrockiana2':0.38269200000000003)68:0.040589999999999904)64:0.032928999999999764)55:0.02569200000000027,('Acanthamoeba castellanii':0.24649600000000005,'Percolomonas cosmopolitus':0.5010529999999997)86:0.10151900000000014)79:0.025370000000000115,((('Emiliania huxleyi2':0.5444819999999999,'Ectocarpus siliculosus2':0.33157099999999984)88:0.04140599999999983,('Phaeodactylum tricornutum2':0.31812299999999993,'Thalassiosira pseudonana':0.3141050000000001)100:0.14312499999999995)83:0.05479300000000009,'Phytophtora ramorum':0.3503750000000001)81:0.023851000000000067)89:0.0504929999999999,'Monosiga brevicollis':0.2860369999999999)100:0.1157849999999998)98:0.10208300000000037,(((((('Nitrobacter hamburgensis':0.3336039999999998,'Rhodobacter sphaeroides':0.43884199999999973)50:0.04724100000000009,'Rhodobacter sphaeroides2':0.29235299999999986)55:0.039915000000000145,'Magnetospirillum magneticum':0.29695000000000027)93:0.05107399999999984,'Burkholderia cenocepacia':0.39371599999999995)97:0.0885180000000001,'Azospirillum sp. B506':0.3747290000000003)100:0.1292089999999999,(((('Vibrio cholerae':0.09468699999999997,'Yersinia pestis':0.11237600000000025)99:0.040904000000000273,'Shewanella baltica':0.13136500000000018)100:0.13906699999999983,('Ralstonia solanacearum':0.16540600000000039,'Verminephrobacter eiseniae':0.29160299999999983)89:0.07348299999999997)90:0.045696999999999655,'Cupriavidus necator':0.3050619999999995)100:0.1527470000000002)93:0.11816100000000018)33:0.02833299999999994,((((('Bacillus anthracis':0.16498200000000018,'Listeria monocytogenes3':0.3305030000000002)86:0.04588800000000015,'Listeria monocytogenes2':0.22628400000000015)86:0.06204199999999993,'Listeria monocytogenes':0.3795010000000003)92:0.05572399999999966,'Bacillus anthracis2':0.34617799999999965)100:0.12485800000000014,('Entamoeba histolytica2':0.004107000000000305,'Entamoeba histolytica1':0.000002)100:0.5960709999999998)46:0.04212000000000016)36:0.07399500000000003,(('Mycobacterium tuberculosis':0.2448769999999998,'Streptomyces coelicolor':0.29817099999999996)92:0.05736000000000008,'Corynebacter diphteriae':0.21103300000000003)99:0.3280829999999999)78:0.12141400000000013,(((((((('Cryptomonas paramecium2':0.23068,'Guillardia theta':0.034340999999999955)80:0.023355000000000015,'Guillardia theta2':0.0442800000000001)81:0.049514999999999976,('Cryptomonas paramecium':0.15079899999999968,'Rhodomonas salina':0.05894599999999972)97:0.01232000000000033)100:0.15977700000000006,'Ectocarpus siliculosus':0.1688550000000002)97:0.04703400000000002,'Aureococcus anophagefferens':0.20622600000000002)66:0.018844000000000083,(('Phaeodactylum tricornutum':0.08674899999999974,'Thalassiosira pseudonana2':0.07185099999999967)100:0.16391800000000023,'Lotharella sp. CCMP6223':0.19770599999999972)55:0.035250000000000004)97:0.0890740000000001,'Emiliania huxleyi':0.3286070000000003)100:0.12284000000000006,((('Euglena gracilis 2':0.06586099999999995,'Euglena longa 2':0.05403400000000014)100:0.21668500000000002,('Eutreptiella gymnastica-like CCMP1594 2':0.1066600000000002,'Eutreptiella gymnastica NIES-381 1':0.20156799999999997)58:0.05191599999999985)100:0.2209080000000001,'Dictyostelium discoideum':0.24817599999999995)100:0.06470500000000001)100:0.31949099999999975)100:1.402419,((((('Homo sapiens':0.033241999999999994,'Mus musculus':0.02125200000000005)100:0.12066700000000008,'Xenopus laevis':0.11681300000000006)99:0.0866889999999998,('Bombyx mori':0.24658399999999991,'Strongylocentrotus purpuratus':0.2650100000000002)100:0.18981000000000003)100:1.0286269999999997,'Methanocaldococcus villosus':0.672304)83:0.07959000000000005,'Picrophilus torridus':0.6835649999999998)61:0.08807000000000009):0.11023249999999996,(('Sulfolobus tokodaii':0.778168,'Thermococcus sp.':0.42450199999999993)100:0.2727170000000001,'Thermoproteus uzoniensis':0.69645)78:0.11023249999999996);

**Triose-phosphate isomerase, dataset listed in Data Set S1, Tab 22:**

((((((((((((((((((('Physcomitrella patens5':0.040072007300000045,'Physcomitrella patens6':0.048183761400000025)100:0.07879999999999998,'Physcomitrella patens4':0.04002912010000004)78:0.02761199999999997,'Physcomitrella patens3':0.07975136779999992)76:0.006335999999999897,'Physcomitrella patens':0.03346880820000009)96:0.10865299999999989,(('Arabidopsis thaliana':0.000002,'Arabidopsis thaliana3':0.000002)100:0.1077539999999999,'Selaginella moellendorffii2':0.07070510409999997)97:0.0735030000000001)96:0.03717999999999999,'Oryza sativa3':0.12602951829999998)100:0.07699600000000006,((((('Chlamydomonas reinhardtii':0.11698240589999997,'Volvox carteri f. nagariensis':0.08515219200000002)100:0.0866499999999999,'Polytomella parva':0.191855286)99:0.03646299999999991,('Dunaliella tertiolecta':0.20556350469999995,'Perkinsus marinus4':0.5401931832)99:0.15029399999999993)97:0.11990999999999996,'Chlorella variabilis2':0.3217202834999999)95:0.02635900000000002,'Coccomyxa subellipsoidea2':0.2604545669)96:0.07412200000000002)84:0.030184999999999906,(((('Entamoeba histolytica':0.43892233699999994,'Bathycoccus prasinos':0.15419648350000004)95:0.06299300000000008,('Micromonas pusilla':0.23001541600000008,'Ostreococcus tauri':0.10614411169999993)95:0.028664000000000023)96:0.09044199999999991,('Pyramimonas amylifera1':0.20381597630000003,'Pyramimonas parkeae2':0.1784122118)99:0.045366999999999935)89:0.009384000000000059,(('Micromonas pusilla2':0.14839228370000002,'Pyramimonas parkeae':0.14279933109999998)99:0.06142099999999995,'Bathycoccus prasinos2':0.21467672680000005)99:0.16389199999999993)96:0.03953999999999991)93:0.05133500000000013,((('Arabidopsis thaliana2':0.07213496639999994,'Oryza sativa':0.06448850579999998)99:0.03091099999999991,'Oryza sativa2':0.0989414340000001)99:0.03819299999999992,('Physcomitrella patens2':0.32979291359999996,'Selaginella moellendorffii':0.15010224169999997)81:0.04843299999999995)100:0.1096410000000001)77:0.048980000000000024,'Gloeochaete wittrockiana':0.38608330420000003)19:0.025167999999999857,((((((('Euglena gracilis 2':0.023077006999999927,'Euglena longa 1':0.030728591599999966)100:0.07364100000000007,('Eutreptiella gymnastica-like CCMP1594 1':0.11235014209999994,'Eutreptiella gymnastica NIES-381 1':0.07029329539999996)98:0.02032400000000001)100:0.1952360000000002,('Cyanidioschyzon merolae':0.3539966353999999,'Rhodella maculata2':0.23339265869999992)43:0.08876000000000017)42:0.026201999999999837,((('Lotharella sp. CCMP6223':0.09814002380000009,'Galdieria sulphuraria':0.3185585583000001)96:0.06917499999999999,'Rhodella maculata':0.40207256069999997)34:0.012383000000000033,('Chondrus crispus2':0.34123839590000005,'Porphyridium aerugineum2':0.1781009121999999)86:0.0819129999999999)82:0.08390000000000009)28:0.024402000000000035,(((('Euglena gracilis 4':0.026999388899999976,'Euglena longa 5':0.036761915600000084)100:0.047997999999999985,('Eutreptiella gymnastica-like CCMP1594 3':0.03674133430000004,'Eutreptiella gymnastica NIES-381 5':0.05712659860000002)99:0.04352)100:0.1037300000000001,(('Eutreptiella gymnastica NIES-381 4':0.05515630789999992,'Eutreptiella gymnastica NIES-381 6':0.0735220970999999)100:0.07766600000000001,'Eutreptiella gymnastica-like CCMP1594 4':0.067144066)100:0.352077)77:0.06706899999999982,((('Euglena gracilis 3':0.013691056999999951,'Euglena longa 3PT':0.04897482080000004)100:0.15859699999999988,'Eutreptiella gymnastica-like CCMP1594 2':0.0974367974999999)100:0.051382999999999956,'Lotharella sp. CCMP6225':0.6945126453000001)82:0.059022999999999826)62:0.04786400000000013)54:0.038270999999999944,((('Euglena gracilis 1':0.01843898910000008,'Euglena longa 2':0.030027032300000034)100:0.02230500000000002,'Euglena longa 4':0.06597307500000005)99:0.023049000000000097,'Euglena deses v. intermedia':0.05461155870000001)100:0.23648099999999994)38:0.037409000000000026,(('Acanthamoeba castellanii':0.8306158663999998,'Monosiga brevicollis':0.21121639430000005)43:0.10932199999999992,'Chondrus crispus':0.23861053359999995)18:0.013222000000000067)28:0.07179699999999989)12:0.011132000000000142,((((('Diplonema papillatum':0.43085338500000003,'Laccaria bicolor':0.23849459410000007)72:0.04758299999999993,'Aspergillus fumigatus':0.2744385118999999)62:0.03388800000000014,'Cryptococcus neoformans':0.3017969917000001)85:0.06045199999999995,'Drosophila melanogaster':0.35438827550000007)75:0.038191000000000086,'Dictyostelium discoideum':0.713396741)64:0.025442999999999882)55:0.014067000000000052,('Chlorella variabilis':0.1905983498999999,'Coccomyxa subellipsoidea':0.22783025169999993)70:0.07641900000000001)80:0.04737500000000017,(((((((((('Eutreptiella gymnastica-like CCMP1594 5':0.07752982419999999,'Eutreptiella gymnastica-like CCMP1594 6':0.06019472460000008)90:0.015066999999999942,('Eutreptiella gymnastica-like CCMP1594 7':0.06719534270000005,'Eutreptiella gymnastica NIES-381 3':0.0692696718000001)95:0.067129)99:0.03776500000000005,'Eutreptiella gymnastica NIES-381 2':0.050784182299999925)100:0.15707300000000002,'Aureococcus anophagefferens':0.20769682890000007)98:0.021999000000000102,'Emiliania huxleyi':0.17970544300000002)100:0.29368799999999995,(('Guillardia theta':0.08098621919999993,'Rhodomonas salina':0.2561899957)100:0.03996299999999997,'Cryptomonas paramecium1':0.242782799)100:0.19305600000000012)98:0.09203899999999998,'Lotharella sp. CCMP6224':0.4555364921)92:0.091148,(('Phaeodactylum tricornutum3':0.24185312790000002,'Thalassiosira pseudonana2':0.16493691519999998)100:0.1435280000000001,'Aureococcus anophagefferens2':0.3566602134000001)93:0.16279399999999988)70:0.02591600000000005,(((('Pythium ultimum var. sporangiiferum':0.08095419809999993,'Pythium ultimum var. sporangiiferum3':0.20297853310000002)71:0.061077999999999966,'Phytophtora ramorum3':0.13733091400000008)86:0.07647800000000005,'Phytophtora ramorum2':0.1149850818)100:0.2534449999999999,'Ectocarpus siliculosus2':0.33480359970000007)76:0.09481099999999998)61:0.037673999999999985,(((('Phaeodactylum tricornutum':0.13851784229999997,'Thalassiosira pseudonana':0.06845136379999994)100:0.2901959999999999,'Ectocarpus siliculosus':0.26026906289999996)39:0.08003699999999991,('Phytophtora ramorum':0.10793930809999996,'Pythium ultimum var. sporangiiferum2':0.10651469689999993)100:0.19541799999999987)93:0.10179000000000027,('Cyanoptyche gloeocystis':0.19865176880000002,'Gloeochaete wittrockiana2':0.20149564460000002)99:0.08707600000000015)66:0.03806699999999985)40:0.019559000000000104)36:0.04318399999999989,(((('Paramecium tetraurelia':0.0400755369000001,'Paramecium tetraurelia3':0.0607650663999999)100:0.18426500000000012,'Tetrahymena thermophila':0.18857421629999993)100:0.15484500000000012,'Paramecium tetraurelia2':0.48158340420000023)91:0.10022799999999998,(('Neospora caninum':0.03950816509999999,'Toxoplasma gondii2':0.012724649599999971)100:0.3850399999999998,'Nannochloropsis gaditana':0.42077524219999995)66:0.028891000000000222)65:0.04786699999999988)81:0.05366800000000005,(((('Perkinsus marinus2':0.019050079699999944,'Perkinsus marinus5':0.005523577700000004)100:0.010491000000000028,'Perkinsus marinus3':0.11278991260000004)99:0.043736999999999915,'Perkinsus marinus':0.07964381480000005)100:0.284983,('Trypanosoma brucei':0.23695351080000004,'Leishmania major':0.1359394801)100:0.306017)81:0.073013)64:0.008229000000000042,(('Lotharella sp. CCMP622':0.6393095537,'Naegleria gruberi':0.29774996259999975)90:0.09457899999999997,'Percolomonas cosmopolitus':0.5301612517999998)84:0.060199000000000114)84:0.08394899999999983,((('Neospora caninum2':0.016546345299999876,'Toxoplasma gondii':0.048956232199999894)100:0.27776899999999993,'Eimeria tenella':0.31343716919999975)100:0.08821600000000007,('Lotharella sp. CCMP6222':0.7955869855,'Porphyridium aerugineum':0.31475295589999996)95:0.07919900000000002)94:0.08510099999999987)100:0.09382050000000008,(((((((('Cryptomonas paramecium3':0.1778660475,'Phaeodactylum tricornutum2':0.5642417883000002)90:0.051026000000000016,'Cryptomonas paramecium2':0.28260288719999993)91:0.12796399999999997,'Pyramimonas amylifera2':0.40851547639999986)100:0.24795100000000003,'Cyanoptyche gloeocystis2':0.47895160429999994)68:0.18542800000000015,('Klebsiella pneumoniae':1.5013004422,'Acinetobacter johnsonii':0.7112480258)51:0.111618)68:0.160204,('Neisseria meningitidis':0.7686049125999999,'Burkholderia thailandensis':0.4484794643000001)68:0.18466000000000005)68:0.07487200000000005,(('Methylobacterium mesophilicum':0.33466249280000016,'Rhizobium tropici':0.5998879656000002)97:0.1312739999999999,'Brevundimonas diminuta':0.553110362)99:0.30996099999999993)74:0.03349000000000002,(('Escherichia coli':0.22351902779999988,'Vibrio azureus':0.2946721084999999)100:0.24208399999999997,'Pseudomonas putida':0.38148796500000004)96:0.07715300000000003):0.09382050000000008);
