# Supplementary material for: The Cryptic Plastid of Euglena longa Defines a New Type of Nonphotosynthetic Plastid Organelle
Source: mSphere. 2020 Oct 21;5(5):e00675-20. doi: 10.1128/mSphere.00675-20 (PMC7580956; doi:10.1128/mSphere.00675-20)
Supplement: FIG S7 [file mSphere.00675-20-sf007.pdf]

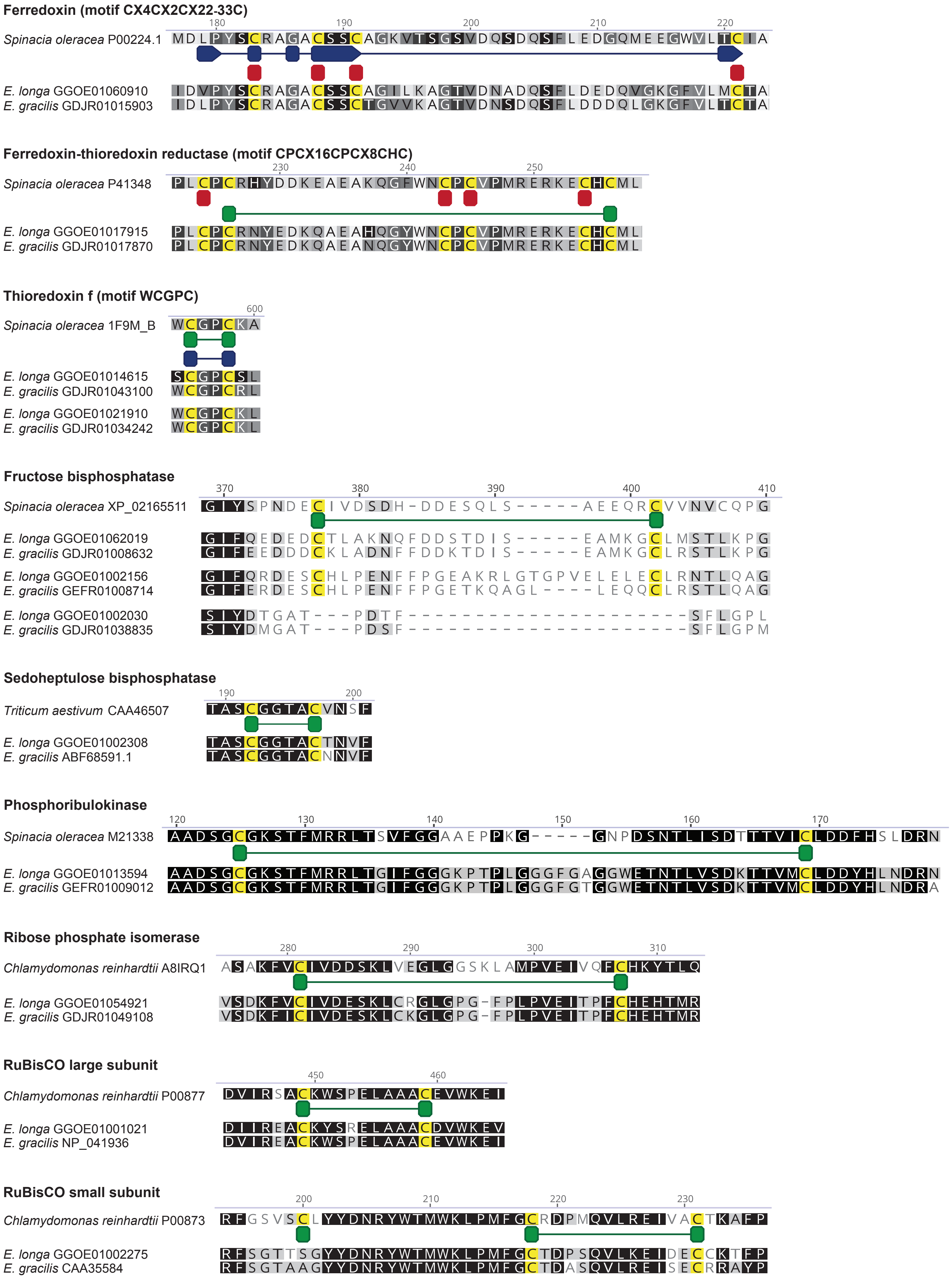
Ferredoxin (motif CX4CX2CX22-33C)
180 190 200 210 220
Spinacia oleracea P00224.1 MDLPYSCRAGACSSCAGKVTSGSVDQSDQSFLEDGQMEEGWVLTCIA
E. longa GGOE01060910 IDVPYSCRAGACSSCAGILKAGTVDNADQSFLDEDQVGKGFVLMCTA
E. gracilis GDJR01015903 IDLPYSCRAGACSSCTGVVKAGTVDNSDQSFLDDDQLGKGFVLTCTA
Ferredoxin-thioredoxin reductase (motif CPCX16CPCX8CHC)
230 240 250
Spinacia oleracea P41348 PLCPCRHYDDKEAEAKQGFWNCPCVPMRERKECHCML
E. longa GGOE01017915 PLCPCRNYEDKQAEAHQGYWNCPCVPMRERKECHCML
E. gracilis GDJR01017870 PLCPCRNYEDKQAEANQGYWNCPCVPMRERKECHCML
Thioredoxin f (motif WCGPC)
600
Spinacia oleracea 1F9M_B WCGPCKA
E. longa GGOE01014615 SCGPCSL
E. gracilis GDJR01043100 WCGPCRL
E. longa GGOE01021910 WCGPCKL
E. gracilis GDJR01034242 WCGPCKL
Fructose bisphosphatase
370 380 390 400 410
Spinacia oleracea XP_02165511 GIYSPNDECIVDSDH-DDESQLS-----AEEQRCVVNVCQPG
E. longa GGOE01062019 GIFQEDEDCTLAKNQFDDSTDIS-----EAMKGCLMSTLKPG
E. gracilis GDJR01008632 GIFEEDDDCKLADNFFDDKTDIS-----EAMKGCLRSTLKPG
E. longa GGOE01002156 GIFQRDESCHLPENFFPGEAKRLGTGPVELELECLRNTLQAG
E. gracilis GEFR01008714 GIFERDESCHLPENFFPGETKQAGL----LEQQCLRSTLQAG
E. longa GGOE01002030 SIYDTGAT---PDTF----------------------SFLGPL
E. gracilis GDJR01038835 SIYDMGAT---PDSF----------------------SFLGPM
Sedoheptulose bisphosphatase
190 200
Triticum aestivum CAA46507 TASCGGTACVNSF
E. longa GGOE01002308 TASCGGTACTNVF
E. gracilis ABF68591.1 TASCGGTACNNVF
Phosphoribulokinase
120 130 140 150 160 170
Spinacia oleracea M21338 AADSGCGKSTFMRRLTSVFGGAAEPKG-----GNPDSNTLISDTTTVICLDDFHSLDRN
E. longa GGOE01013594 AADSGCGKSTFMRRLTGIFGGGKPTPLGGGFGAGGWETNTLVSDKTTVMCLDDYHLNDRN
E. gracilis GEFR01009012 AADSGCGKSTFMRRLTGIFGGGKPTPLGGGFGTGGWETNTLVSDKTTVMCLDDYHLNDRA
Ribose phosphate isomerase
280 290 300 310
Chlamydomonas reinhardtii A8IRQ1 ASAKFVCIVDDSKLVEGLGGSKLAMPVEIVQFCHKYTLQ
E. longa GGOE01054921 VSDKFVCIVDESKLCRGLGPG-FPLPVEITPFCHEHTMR
E. gracilis GDJR01049108 VSDKFICIVDESKLCKGLGPG-FPLPVEITPFCHEHTMR
RuBisCO large subunit
450 460
Chlamydomonas reinhardtii P00877 DVIRSACKWSPELAAACEVWKEI
E. longa GGOE01001021 DIIREACKYSRELAAACDVWKEV
E. gracilis NP_041936 DVIREACKWSPELAAACEVWKEI
RuBisCO small subunit
200 210 220 230
Chlamydomonas reinhardtii P00873 RFGSVSCLYYDNRYWTMWKLPMFGCRDPMQVLREIVACTKAFP
E. longa GGOE01002275 RFSGTTSGYYDNRYWTMWKLPMFGCTDPSQVLKEIDECCKTFP
E. gracilis CAA35584 RFSGTAAGYYDNRYWTMWKLPMFGCTDASQVLREISECRRAYP
